# New pteridophyte species and combinations from the Marquesas Islands, French Polynesia

**DOI:** 10.3897/phytokeys.4.1602

**Published:** 2011-07-12

**Authors:** David H. Lorence, Warren L. Wagner, Kenneth R. Wood, Alan R. Smith

**Affiliations:** 1 National Tropical Botanical Garden, 3530 Papalina Road, Kalaheo, HI 96741 USA; 2 Department of Botany, MRC-166, National Museum of Natural History, Smithsonian Institution, P.O. Box 37012, Washington, DC 20013-7012; 3 1001 Valley Life Sciences Building, no. 2465, University Herbarium, University of California, Berkeley, CA 94720–2465

**Keywords:** *Blechnum*, *Cyclosorus*, *Dryopteris*, French Polynesia, Marquesas Islands, *Polystichum*, pteridophytes, *Pteris*, *Thelypteris*

## Abstract

Intensive botanical exploration of the Marquesas Islands (French Polynesia) for the Vascular Flora of the Marquesas Islands and Flore de la Polynésie française projects has resulted in numerous additional new collections from these islands. Study of these collections has brought to light 11 new species of pteridophytes (ferns and lycophytes) which are described herein: *Blechnum pacificum* Lorence & A. R. Sm., **sp. nov.**, *Cyclosorus castaneus* A. R. Sm. & Lorence, **sp. nov.**, *Cyclosorus florencei* A. R. Sm. & Lorence, **sp. nov.**, *Dryopteris macropholis* Lorence & W. L. Wagner, **sp. nov.**, *Dryopteris sweetorum* Lorence & W. L. Wagner, **sp. nov.**, *Polystichum kenwoodii* Lorence & W. L. Wagner, **sp. nov.**, *Polystichum uahukaense* Lorence & W. L. Wagner, **sp. nov.**, *Pteris hivaoaensis* Lorence & K. R. Wood, **sp. nov.**, *Pteris marquesensis* Lorence & K. R. Wood, **sp. nov.**, *Pteris tahuataensis* Lorence & K. R. Wood, **sp. nov.**, and *Thelypteris marquesensis* Lorence & K. R. Wood, **sp. nov.** One new combination is made: *Cyclosorus marquesicus* (Holttum) Lorence & A. R. Sm., **comb. nov.** (based on *Plesioneuron marquesicum* Holttum). An analysis of the conservation status of these new Marquesas Islands taxa reveals they are in need of inclusion in the IUCN Red List with conservation status ranging from vulnerable (one species), and endangered (four species), to critically endangered (five species).

## Introduction

The only recent treatment of the Marquesan pteridophytes (=ferns and lycophytes, used interchangeably herein) is Forrest and Elizabeth Brown’s Flora of Southeastern Polynesia(Brown & Brown 1931). This is essentially a report on the plants collected by the Browns on the Bayard Dominick Expedition (1921–1922), by E. H. Quayle, B. W. Jones, and R. Beck on the Whitney Expedition (1921–1922), and by the Pacific Entomological Survey (1929–1932) (Lorence and Wagner 1997). The Browns’ flora did not include earlier collections not then represented in the Bishop Museum, except as they may have been recorded in the French Polynesian flora of Drake del Castillo (1892). In his Flore de la Polynésie Française Drake del Castillo recognized 142 fern and lycophyte species from the region, three of which were described as new. Brown and Brown (1931) recorded 103 species from southeastern Polynesia, or more specifically 122 taxa including varieties and forms of which 59 (48%) were described as new. Although the Browns’ work suffers from a number of shortcomings such as non-parallel descriptions, atypical typifications, and errors in identification, it has remained the only modern pteridophytes flora available for the Marquesas.

Copeland (1932) subsequently published a floristic treatment of the Society Islands pteridophytes that included 164 species from this archipelago, but did not include the Marquesas Islands. Most recently, Kato et al. (2008) published an illustrated flora of ferns and lycophytes of the South Pacific islands, encompassing Samoa, Fiji, Vanuatu, and New Caledonia. Revisions of only a few taxonomic groups occurring in French Polynesia and the Marquesas have been published to date, e.g. *Elaphoglossum* (Rouhan et al. 2008) and Hymenophyllaceae (Ebihara et al. 2006). Useful identification guides include an online key and pictorial checklist of the pteridophytes of Moorea, available at http://ucjeps.berkeley.edu/moorea/pteridophytes.html. A comprehensive published treatment of Marquesan pteridophytes is clearly needed.

This paper increases our knowledge of the Marquesan pteridophyte flora by describing 11 new species and proposing one new combination. The present contribution forms part of a series of precursor publications with the goal of producing the first complete Vascular Flora of the Marquesas Islands (Lorence and Wagner 1997). Currently available online as a Web-based flora with a searchable database of descriptions, photos, literature and specimens, it may be accessed at http://botany.si.edu/pacificislandbiodiversity/marquesasflora/index.htm. This project will provide a comprehensive treatment of all Marquesan pteridophytes, for which the essentially completed treatment is now available on the website. A primary goal is to publish the Vascular Flora of the Marquesas Islands as a two volume printed work. A second goal involves collaboration on the Flora of French Polynesia project headed by Jacques Florence under the auspices of the French Institute pour la Recherche et Developpement (IRD, formerly ORSTOM). To date only two volumes of the modern *Flore de la Polynésie Française* have been published (Florence 1997, 2004), although Dr. Florence is currently finalizing a field guide to the French Polynesian pteridophytes.

## Island Names

Orthographic variation exists for certain of the Marquesas Islands. For the sake of consistency we herein utilize the names accepted by the French Polynesian Government (see website at: www.presidence.pf) for the islands. In the following list accepted names are in boldface and alternative spellings are listed in parentheses: **Fatu Hiva** (Fatuhiva, Fatu Iva), **Hiva Oa** (Hivaoa), **Mohotani** (Motane), **Nuku Hiva** (Nukuhiva), **Tahuata, Ua Huka** (Uahuka), and **Ua Pou** (Uapou).

## Conservation Status

As the Marquesan environment is under serious threat from human impacts, feral animals, and weeds (Florence and Lorence 1997) the conservation status was estimated for each newspecies. When evaluated using the IUCN criteria for endangerment (IUCN 2001, see also www.iucnredlist.org/info/categories_criteria2001), all but one of the new Marquesan pteridophyte species fall into the Endangered (EN) or Critically Endangered (CR) categories, which designates species facing the highest risk of extinction in the wild. One species, *Blechnum pacificum* which occurs on five Marquesan islands, three Society Islands, and Rapa Iti in the Austral Islands, is considered Vulnerable (VU). The IUCN **Endangered** (EN) criteria include: B1having known ranges less than 5000 km2; B2 an area of occupancy of less than 500 km2; a, b severely fragmented or known to exist at no more than five locations; c continuing decline in the quality of habitat; or D, a populations size less than 250 mature individuals. The IUCN **Critically Endangered** (CR) criteria include: B1 having known ranges of less than 100 km2 and/or B2, an area of occupancy of less than 10 km2; D, population size of less than 50 mature individuals; and estimates including at least two of the following: a, severely fragmented or known to exist at only a single location; b, continuing decline in the extent and/or quality of habitat, extent of occurrence or occupancy, or number of mature individuals.

## Methodology

All measurements given herein are taken from dried herbarium specimens, although certain features such as shapes were supplemented with information from field notes and photos. Measurements are presented in the descriptions as follows: length × width, followed by units of measurement (mm or cm). All specimens cited in this paper have been seen by the authors. Specimens from the following herbaria were studied: AD, BISH, BR, K, MO, NY, P, PAP, PTBG, and US. The area of occupancy (distribution) was calculated using herbarium collection data and field observations, and the conservation status is proposed following the IUCN Red List Category criteria (IUCN 2001; www.iucnredlist.org/info/categories_criteria2001).

## Systematics

### BLECHNACEAE

#### Blechnum

*Blechnum* L. is a cosmopolitan genus of about 180 species especially well represented in the southern hemisphere (Mabberley 2008). Brown and Brown (1931) recorded three species from the Marquesas Islands: the endemic *Blechnum nukuhivense* E. D. Br., the rather widespread Pacific species *Blechnum vulcanicum* (Bl.) Kuhn, and *Blechnum capense* (L.) Schltdl. However, it is apparent that the latter name has been misapplied to Polynesian plants, which we describe below as a new species. The three Marquesan *Blechnum* can be separated by the following key.

Key to *Blechnum* in the Marquesas Islands

**Table:** 

1a	Sterile fronds 1-pinnate, nearly all the pinnules contracted at their bases and free from rachis, the apex with a conform terminal pinna similar to lateral pinnae	*Blechnum pacificum*
1b	Sterile fronds entire, pinnatifid, or pinnatisect with the segments adnate and not contracted at their bases, or rarely the basal segments pinnate and the basiscopic base contracted and not adnate, the apex lacking conform terminal pinna	2
2a	Sterile fronds entire or sometimes irregularly pinnatifid toward middle and base	*Blechnum nukuhivensе*
2b	Sterile fronds pinnatisect or rarely the basal segments pinnate	*Blechnum vulcanicum*

##### 1. Blechnum pacificum

Lorence & A. R. Sm. sp. nov.

urn:lsid:ipni.org:names:77112673-1

http://species-id.net/wiki/Blechnum_pacificum

Figs 1 2 14A 

###### Latin.

Species Blechno venoso Copel. affinis, sed stipitis squamis non tan numerosis, deciduis, leviter castaneis usque ad castaneis, sterilibus pinnis cum axialibus glabris et paulatim prominentibus venis, fertilibus pinnis cum viridi expansa textura prope basin differt.

###### Type.

**Marquesas Islands:** Ua Pou: Poumaka Summit Trail, 690 m elevation, 9°23'33"S, 140°04'59"W, 19 June 2004, L. M. Dunn and D. H. Lorence 481 (Holotype: PTBG-041866!, PTBG-041867! [2 sheets]; Isotypes BISH!, P!, PAP!, US!).

*Blechnum capense* sensu E. D. Br. & F. B. Br., non Burm. f., non (L.) Schltdl.

###### Description.

*Large terrestrial ferns*; *rhizomes* erect or suberect, rarely decumbent and dorsiventral, short, stout, (10–)15–20 mm in diameter (excluding scales), apex covered with scales; scales of rhizome and bases of stipes linear-subulate to oblong-ovate, 20–30 ×1–3(–5) mm, thin, light brown to brown, concolorous but somewhat thicker and darker basally and in center, basifixed, base rounded or truncate, apex sinuate, margins entire or subentire, cells linear, arranged in vertical rows. *Fronds* clustered near apices of rhizomes, usually dimorphic but both fertile and sterile pinnae occasionally occurring on the same blade; stipes 41–102 cm long, 4–10 mm in diameter, stout, light to dark brown when fresh, drying stramineous, densely scaly toward the bases, sparsely so distally, eventually glabrescent, smooth, grooved adaxially. *Sterile blades* 1-pinnate, oblong-ovate to oblong-elliptic, (35–)50–100 × (20–)30–60 cm, coriaceous to subcoriaceous, stiff, rachises stramineous or sometimes dark brown, proximal pinnae only slightly or not at all reduced, apices acute, each blade with a conform terminal pinna, when young the rachises, costae, costules, and veins scaly with thin, scurfy, brown to pale brown, oblong-ovate to subulate, contorted-sinuate scales to 10 mm long with margins subentire or sometimes lacerate basally; pinnae 19–30 on a side, with a slightly swollen, mammiform aerophore at the base each pinna, basal pinnae opposite and shortly stipitate (to 2 mm), the distal ones becoming subopposite to alternate, sessile with basiscopic base often adnate to rachis, terminal pinna free, conform, medial pinnae 11–30 × 1.5–2.5 cm, linear, margins serrulate, often decidedly undulate, apices acute or acuminate, serrulate, acroscopic bases oblique-cuneate, basiscopic base truncate or rounded, the veins prominulous, simple or 1-forked, free, each ending in a marginal tooth; *fertile fronds* subequal to or slightly smaller than sterile, with up to 40 pinnae pairs (sometimes the proximal pinnae sterile and distal pinnae fertile on the same frond), fertile pinnae approximately the same length as sterile pinnae but narrower, 2.5–5 mm wide, the margins strongly revolute, the sori covering most of the abaxial surface but usually with some expanded green tissue at the base, adaxially glabrate. *Sori* linear, abaxial surface of pinnae bordered or partly covered by the reflexed, scarious, erose blade margins except on expanded green bases. *Spores* subellipsoidal, 67 × 47 µm including a perispore 8–12 µm wide.

**Figure 1.**
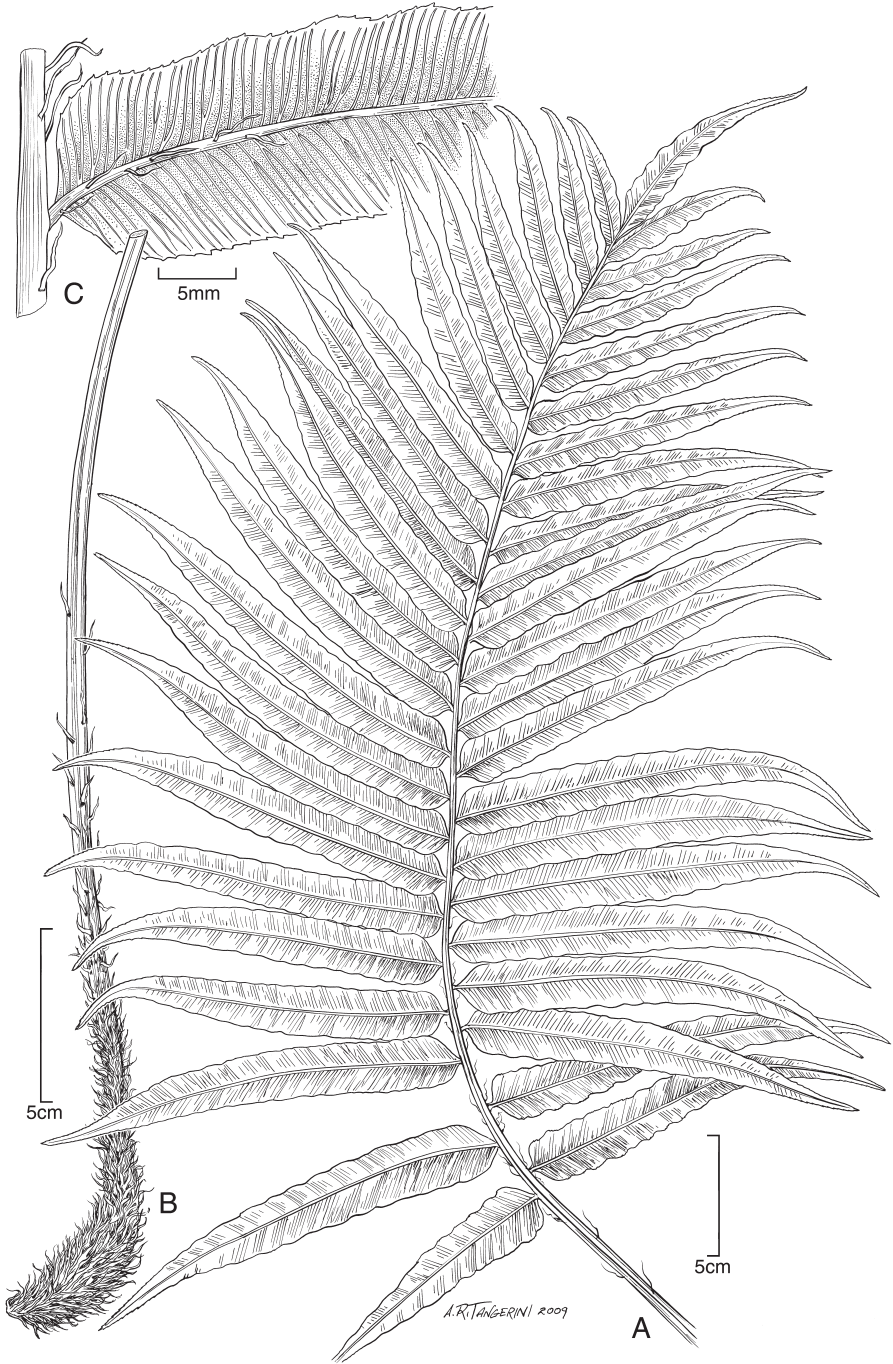
*Blechnum pacificum* Lorence & A. R. Sm. **A** sterile frond, blade **B** sterile frond, stipe **C** sterile frond, pinna base. Drawn from the type collection (Dunn and Lorence 481).

**Figure 2.**
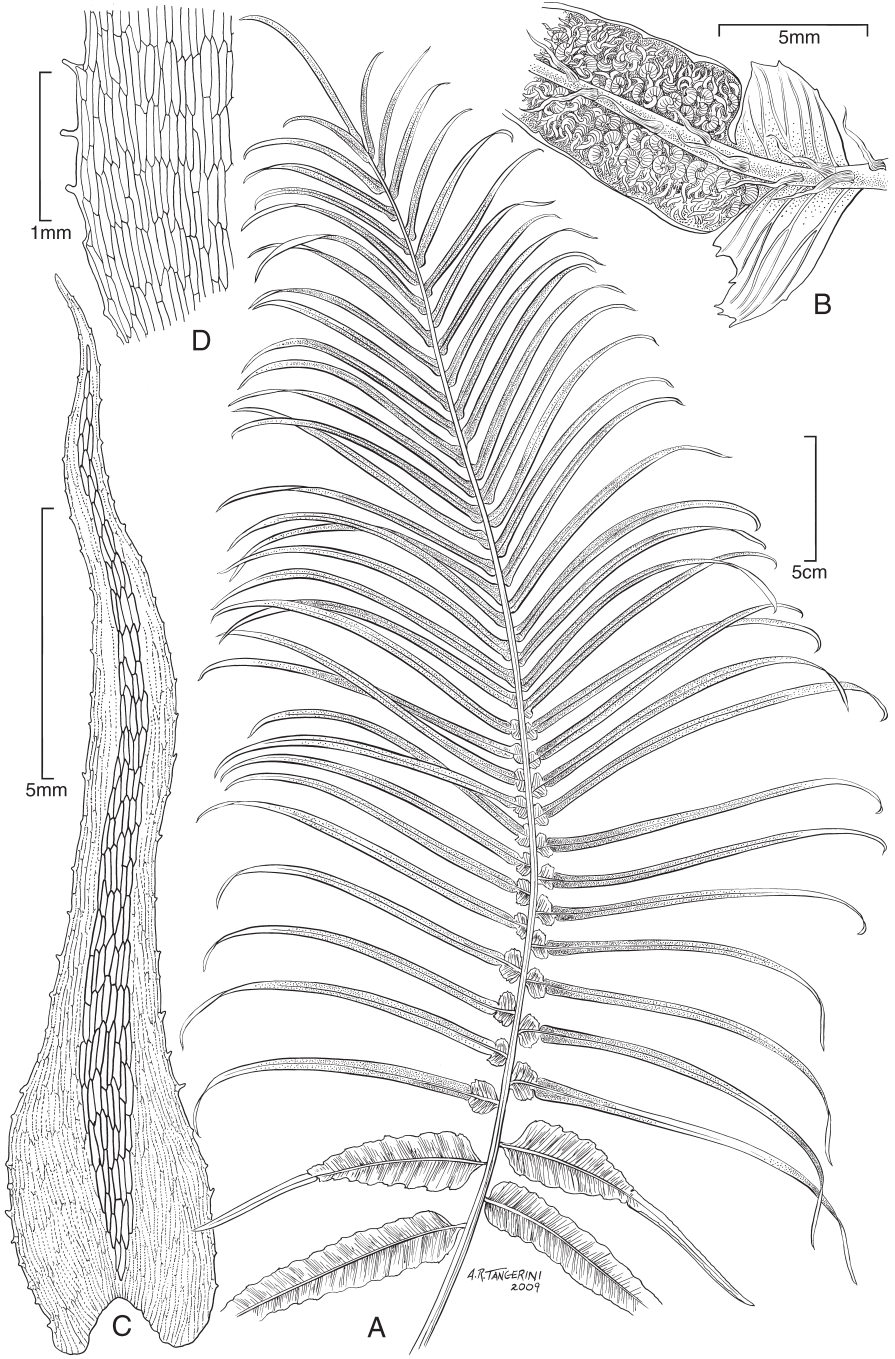
*Blechnum pacificum* Lorence & A. R. Sm. **A** fertile frond, blade **B**, fertile frond, pinna base showing expanded sterile portion **C** scale from base of stipe **D** part of scale from base of stipe, detail of cells. Drawn from the type collection (Dunn & Lorence 481).

###### Distribution.

Known from the Marquesas Islands (Nuku Hiva, Ua Pou, Hiva Oa, Tahuata, and Fatu Hiva), Society Islands (Moorea, Raiatea, and Tahiti), and Austral Islands (Rapa Iti).

###### Ecology.

This new terrestrial species occurs in clearings or shade from 380 to 1500 m elevation in lowland to montane mesic and wet forests, shrubland or fernland, in valleys, on slopes or ridge crests and rocky banks. In the Marquesas associated species include *Crossostylis biflora* J. R. Forst. & G. Forst., *Dicranopteris linearis* (Burm. f.) Underw.*, Freycinetia impavida* (Gaudich. ex Hombr.) B. C. Stone*, Metrosideros collina* (J. R. Forst. & G. Forst.) A. Gray*, Pandanus tectorius* Parkinson*, Santalum insulare* Bertero ex A. DC., and other native tree, shrub, and pteridophyte species. Threats include competition from alien plant species and damage from feral ungulates. Although this is the most widely distributed of the new species, it is clearly at risk due to habitat loss and degradation.

###### Etymology.

The epithet refers to the Pacific distribution of this new species.

###### Conservation status.

IUCN Red List Category: **Vulnerable** (VU): B1: total area of occupancy less than 20,000 km2 (ca. 750 km2); b (i–iii), habitat continuing decline inferred; B2: total area of occupancy less than 2000 km2 (ca. 750 km2); B2b (i–iii), habitat continuing decline inferred. The suitable habitat for *Blechnum pacificum* on most islands of occurrence is indicated as a declining or endangered environment, threatened by human activity (deforestation, fire), feral animals, and invasive plants, reducing the extent of the forest.

###### Specimens examined.

**Austral Islands: Rapa Iti.** Perau–Mamuere summit, eastern peaks, 27°S, 144°W, 10–100 ft (3–30 m), Wood & Faraire 9777 (NY, PTBG [4 sheets]), Fosberg 11567 (UC), Fosberg 11601 (UC), Fosberg 11605 (UC); St. John & Fosberg 15293 (UC). **Society Islands:** **Moorea:** Mt. Rotui, 149°50'18W,  17°30'38S, 916 m, Nitta & Vinette 212 (PAP, UC); Mt. Tohiea, 149.819°, 17.553°, 994 m, Nitta & Vinette 320 (PAP, UC). **Tahiti.** Pirae–Maoua Aorai trail, Quayle 105A (BISH, UC); Orofena, east side of south ridge, ravine in rain forest, St. John & Fosberg 17114 (BISH); Mt. Marau road, crest between Tapaerui and Punaruu valley, 1250 m, Fosberg 62647 (PTBG [2 sheets], US); Orofena, south ridge, moist thicket on exposed ridge, 1600 m, St. John & Fosberg 17082 (UC); Mahina, Ahonu-tuauru, 2875 ft (876 m), Grant 4394 (UC); Aorai, in summit shrubs, 6700 ft (2042 m), M. L. Grant 3791 (UC); Faaa, Mt. Marau, 3 km below TV tower, 1300 m, Hodel 1375 (UC); Fautaua, below Diadem, 2830 ft (863 m), Grant 3546 (UC). **Raiatea:** Temehani Plain, Moore 175 (BISH [2 sheets]). **Marquesas Islands:** **Nuku Hiva:** Toovii, 1000 m, Brown & Brown 528 (BISH); Toovii Plateau, spur of Mt. Ooumu, 790 m, Gagné 1039 (US); Tauamaka, Toovii plateau, 1000 m, Mumford & Adamson 576 (BISH, UC); without precise locality, 1000 m, Quayle 1284 (BISH), Quayle 1298 (BISH). **Ua Pou:** Mt. Tekahoipu, 800 m, Quayle 1138 (BISH [2]); Tekohepo, summit, 2500–3000 ft (762–914 ft), 09°24'31"S, 140°04'21"W, Wood & Perlman 6455 (PTBG [2 sheets], US); Drainage northwest of Teavahaakiti, 700 m, Wood 10458 (P, PAP, PTBG, US). **Hiva Oa:** road from Atuona to Puamau, just below Ootua, 625–700 m, Sachet & Decker 1904 (PTBG, US); Puamau, along Puamau–Atuona trail, 500–650 m, Decker 1190 (PTBG, US); Feani, 800 m, Brown 877 (BISH [3]); Atuona–Feani Trail, ridge crest, 1200–1300 m, 24–26 Sep. 1963, Sachet & Decker 1160 (US [2]); Montagnes NW du Temetiu, entre la haute vallée de Hanamenu et la crête de Temetiu–Feani, 850 m, Schäfer 5932 (US); Vaiata, NW slopes of Mt. Ootua, 800 m, Mumford & Adamson 355 (BISH); Feani ridge to upper slopes of dry side of island, 1150 m, Oliver & Schäfer 3130 (US [2]); Temetiu, 1100 m, Pacific Entomol. Surv. 156 (BISH); above Atuona, 800 m, Pacific Entomol. Surv. Ex 355 (BISH); Hanaiapa, 700 m, Jones 1613 (BISH [2 sheets]); without precise locality, 800 m, Brown 16 (BISH). **Tahuata:** de Hamatea [Amatea] à la crête centrale de l’île, 750–850 m, Thibault 64 (US); Mt. Amatea, 1000 m, Jones 1796 (BISH). **Fatu Hiva:** ‘Omo’a–Ouia–Mounanui Trail, 690 m, Gagné & Montgomery 2323 (BISH); trail from ‘Omo’a along Punaitai ridge crest to base of Tekou peak, 550–840 m, Lorence et al. 6171 (BISH, PAP, PTBG); Hanavave, 600 m, Jones 1826 (BISH); Sentier d’Ouia, W du col, lieu-dit Tahuna, 620 m, Schäfer 5803 (US).

###### Discussion.

The name *Blechum capense* Burm. f. has been erroneously applied to this new Polynesian species (Brown and Brown 1931; Copeland 1932). The true *Blechnum capense* is confined to southern Africa and some nearby islands (Burrows 1990; Roux 2001). This southern African species has also been treated under the name *Blechnum sylvaticum* Schelpe (Schelpe 1979; Jacobson 1983), on the false assumption that the type of *Blechnum capense* Burm. f. was a mixed collection of two species (Jacobson 1983). However, there seem to be good reasons for placing *Blechnum sylvaticum* in synonymy under *Blechnum capense* (Roux 1982; Schelpe and Anthony 1986; Burrows 1990; Roux 2001).

The name *Blechnum procerum* G. Forst. has also been incorrectly applied to the Polynesian plants. Quoting Nicolson and Fosberg (2004: 125–126), who stated that the type of *Blechnum procerum* is from New Zealand: “Tindale (1960b: 254) published a photo (t. 7) of the Goettingen material [Nova Zeelandia, *Forster 295*, GOET] as ‘Type specimen’ and referred to BM and K specimens as ‘Forster material.’ Chambers and Farrant (1998a: 4), without discussion, said ‘T. Noua Zeelandia, Forster; lecto (here chosen): K; isolecto: BM, GOET (photo seen).’” Nicolson and Fosberg (2004: 126) further stated “There is considerable confusion about the names applied to this taxon and the following quotation from Brownsey and Smith-Dodsworth (2001) summarized it well: ‘The name *Blechnum minus* was used incorrectly by Allan (1961) and Crookes (1963) for the plant we call *Blechnum procerum*. The true *Blechnum minus*, or swamp kiokio, is only doubtfully distinct from the common kiokio (previously known as *Blechnum capense*).’”  *Blechnum procerum* has lower pinnae only slightly reduced (not less than half the length of the median pinnae), fertile fronds are up to 50% longer than sterile fronds, and the fertile pinnae show no expanded green tissue at the base in the type as in *Blechnum pacificum*. Clearly, the name *Blechnum procerum* does not apply to the Polynesian plants.

In New Zealand, closest relatives appear to be *Blechnum novae-zelandiae* T. C. Chambers & P. A. Farrant and *Blechnum minus* (R. Br.) Ettingsh., neither of which is conspecific with our Polynesian plants. *Blechnum novae-zelandiae* is superficially similar but differs in having reduced proximal pinnae and distinctive “black-spot” rhizome and stipe scales with dark brown or black centers and pale margins (Chambers and Farrant 1998). From evidence presented in a recent paper on the phylogeny of New Zealand Blechnaceae by Shepherd et al. (2007, Fig. 2), it seems likely that if *Blechnum pacificum* were sampled, it would fall somewhere in the clade containing *Blechnum wattsii* Tindale and *Blechnum novae-zelandiae*.

Several species from New Caledonia, all considered endemic, form a confusing array of species somewhat similar to *Blechnum pacificum*. These include *Blechnum confusum* (E. Fourn.) Brownlie, *Blechnum chauliodontum* Copel., and *Blechnum subcordatum* (E. Fourn.) Brownlie. Brownlie’s (1969) illustration and characterization of *Blechnum subcordatum* suggests that it differs in having smaller fronds with less scaly stipes and rachises, fewer pinnae pairs (5–15), and sterile pinnae not so undulate at the margins. *Blechnum confusum* differs in its strongly ascending, more sharply serrulate and less scaly pinnae (sterile blades are nearly glabrous). The closest species in Malesia (excluding Papua New Guinea) appears to be *Blechnum vestitum* (Blume) Kuhn, nom. cons., non *Blechnum vestitum* T. Moore (see Chambers and Farrant 2001; Chambers 2004; McNeill et al. 2006: 438). The Papua New Guinea species *Blechnum dilatatum* (Brause) T. C. Chambers & P. A. Farrant is similar to *Blechnum pacificum* in having fertile pinnae with an expanded basal sterile region, but in the latter the margins are never revolute and the rhizome scales are thin, concolorous, and pale to medium brown. No names of taxa with types from Fiji apply to the new species, the closest species there being *Blechnum milnei* (Carruthers) C. Chr. (historically also called *Blechnum procerum*), which differs in having very large fronds with generally broader, less coriaceous pinnae and a less scaly rachis and costae on the sterile blades, and fertile pinnae lacking expanded green tissue at the base.

Among the Polynesian species of *Blechnum*, *Blechnum pacificum* seems most closely related to *Blechnum venosum* Copel. from Rapa Iti in the Austral Islands. In addition to having copious, shiny, dark brown, almost blackish scales on the stipes as noted in the diagnosis, the veins of *Blechnum venosum* are very prominent and strongly raised above the surface on the abaxial side of blades, whereas the veins in *Blechnum pacificum* are visible abaxially but scarcely, if at all, raised.  This gives *Blechnum venosum* a much harsher, more cartilaginous appearance. Also, in *Blechnum venosum*, there are very short hairs on the veins abaxially and some hairs are even present between the veins on laminar tissue, but *Blechnum pacificum* lacks such hairs. In *Blechnum venosum*, some of these hairs on the veins and laminar tissue appear multicellular (septate, but uniseriate), and glandular or gland-tipped. Also, pinna margins in *Blechnum pacificum* are more crenulate (scalloped) than in *Blechnum venosum* which has entire margins.

### DRYOPTERIDACEAE

#### Dryopteris

*Dryopteris* Adanson is a large, essentially cosmopolitan genus of around 225 species with its greatest diversity in north temperate regions (Fraser-Jenkins 1986a, 1989; Mickel and Smith 2004). Previously only a single species, *Dryopteris fatuhivensis* E. D. Br., was recorded from the Marquesas (Brown and Brown 1931), where it occurs on Nuku Hiva, Fatu Hiva, and Ua Huka. *Dryopteris fatuhivensis* was placed in subg. *Dryopteris*, sect. *Hirtipedes* by Fraser-Jenkins (1986b) along with related species from Asia, including the widespread *Dryopteris hirtipes* (Blume) Kuntze which includes two subspecies, subsp. *hirtipes* and subsp. *atrata* (Kunze) Fraser-Jenk., both in southeast Asia. We follow Fraser-Jenkens (1989) in recognizing *Dryopteris fatuhivensis* as a Marquesan endemic species distinct from *Dryopteris hirtipes*.

Recent collections from the Marquesas have revealed the presence of two additional, distinctive endemic *Dryopteris* species distinguished by their large, 3-pinnate to 3-pinnate-pinnatifid fronds. Both species have normally developed spores and lack any morphological features suggesting hybrid origin. Morphologically they are quite different than *Dryopteris fatuhivensis*, from which they may be separated by the characters in the following key.

Key to *Dryopteris* in the Marquesas Islands

**Table:** 

1a	Blades 1-pinnate to 1-pinnate-pinnatifid	5–10 mm distant with margins serrate, segments acute at apex	*Dryopteris fatuhivensis*
1b	Blades 3-pinnate to 3-pinnate-pinnatifid	2
2a	Ultimate pinnule segments spaced 5–10 mm distant with margins serrate, segments acute at apex	*Dryopteris sweetorum*
2b	Ultimate pinnule segments spaced 3–5 mm distant, margins crenate or lobed ¼ –2/3 toward costule, segments truncate or obtuse at apex	*Dryopteris macropholis*

##### 2. Dryopteris macropholis

Lorence & W. L. Wagner sp. nov.

urn:lsid:ipni.org:names:77112674-1

http://species-id.net/wiki/Dryopteris_macropholis

Figs 3 4 14B, C, D 

###### Latin.

A ceteris marchinonicis speciebus integra margine maximis usque ad 80 × 14 mm squamis (vel paleis) vestitis rhizomate atque stipitis base, ampla tripinnati-pinnatifida lamina, ultimis usque ad 12–19 × 4–8 mm inferne glabris pinnulis, truncatis vel crenatis, 1/4–2/3 costa dissectis lobis, sparsis parvis castaneis paleis vestita rhachidi, quaque pinnula 1–4 indusiatorum sororum paribus munita, glabro indusio, praecipue differt.

###### Type.

**Marquesas Islands**: Ua Huka: Hitikau region, ascended via Matukuoha ridge overlooking Hane, constitutes the summit of the single crater of Ua Huka, 700 m, UTM 0661697–9015668, 5 Dec 2003, K. R. Wood 10489 (Holotype PTBG-041629!, PTBG-041630! [2 sheets]; Isotypes P!, PAP!, US!).

###### Description.

*Terrestrial ferns*; *rhizomes* suberect, 20–25 cm long, 5–7 cm in diameter (to 15 cm including scales), densely clothed with pale brown to reddish brown or dark brown scales; scales of rhizome and base of stipe (10–)20–80 × (1–)2–5–14 mm, thin, narrowly oblong-elliptic to linear-lanceolate, falcate, usually twisted distally, concolorous, lustrous, medium to dark brown or reddish brown, margins entire, cells narrowly rectangular to linear-fusiform. *Fronds* clustered, 5–7 per rhizome, erect-arching; stipes (35)49–75 cm long, 4–6 mm in diameter medially, about as long as the blades, adaxially grooved, reddish brown to stramineous, entire length clothed in dense, persistent, spreading, lustrous, light to dark brown or reddish brown, linear-oblong to linear-lanceolate twisted scales to ca. 20 × 3 mm, margins entire or subentire and fringed with short-stipitate glands, bases darkened at point of attachment, mixed with smaller bristlelike and hairlike scales, surfaces bearing short, gland-tipped hairs, scales progressively smaller and finer distally and on rachis, stipes of older fronds punctate with dark scale bases; *blades* thickly chartaceous, dark above green when fresh, paler beneath, 50–100 × 32–66 cm, ovate-deltate, 3-pinnate to 3-pinnate-pinnatifid at least in lower half, distally mostly 2-pinnate-pinnatifid; rachises stramineous to light brown, densely scaly with persistent medium to dark brown, spreading bristlelike scales to 9 × 1 mm, margins entire or with sparse sessile glands, mixed with short glandular hairs, rachises of older fronds punctuate with dark brown scale bases; pinnae opposite to subopposite, (11–)13–20 on a side, spreading, ovate-oblong to linear-oblong, apex acuminate, lowermost pinnae the largest, 20–33 × 11–19 cm, with 11–16 pairs of pinnules, slightly inequilateral, basiscopic basal pinnules 7–10.5 cm long, acroscopic basal pinnules shorter, 4–8.5 cm long, lowermost pinnules usually the largest, distal pinnae stalked 3–6 mm becoming sessile, apices pinnatifid; largest ultimate pinnules 12–19 × 4–8 mm, spaced 5–10 mm distant, obtuse to truncate at apices, margins crenate or cut ca. ¼ –2/3 toward costule, adaxially glabrous, abaxially glabrous except for scattered small, spreading, brown linear scales on rachises; veins forking 1–2 times, scarcely visible to visible on both surfaces, depressed adaxially and prominulous abaxially; each fertile pinnule usually with 1–4 pairs of inframedial sori. *Sori* with indusia 0.4–0.6 mm in diameter, brown, thick, glabrous. *Spores* dark brown.

**Figure 3.**
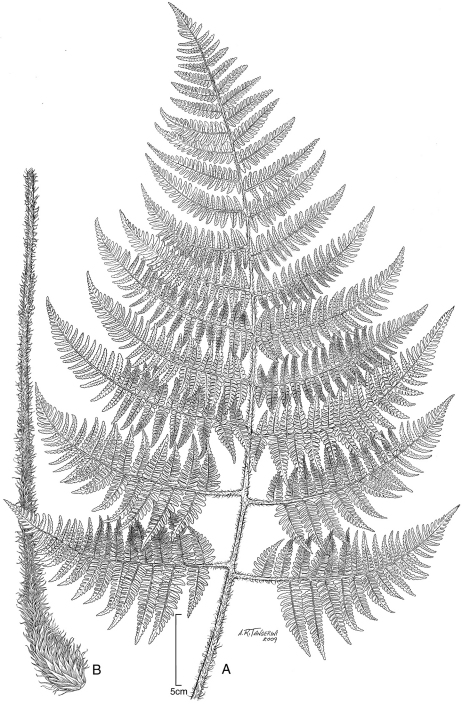
*Dryopteris macropholis* Lorence & W. L. Wagner. Frond with **A** blade and **B** stipe. Drawn from the type collection (Wood 10489) and field images.

**Figure 4.**
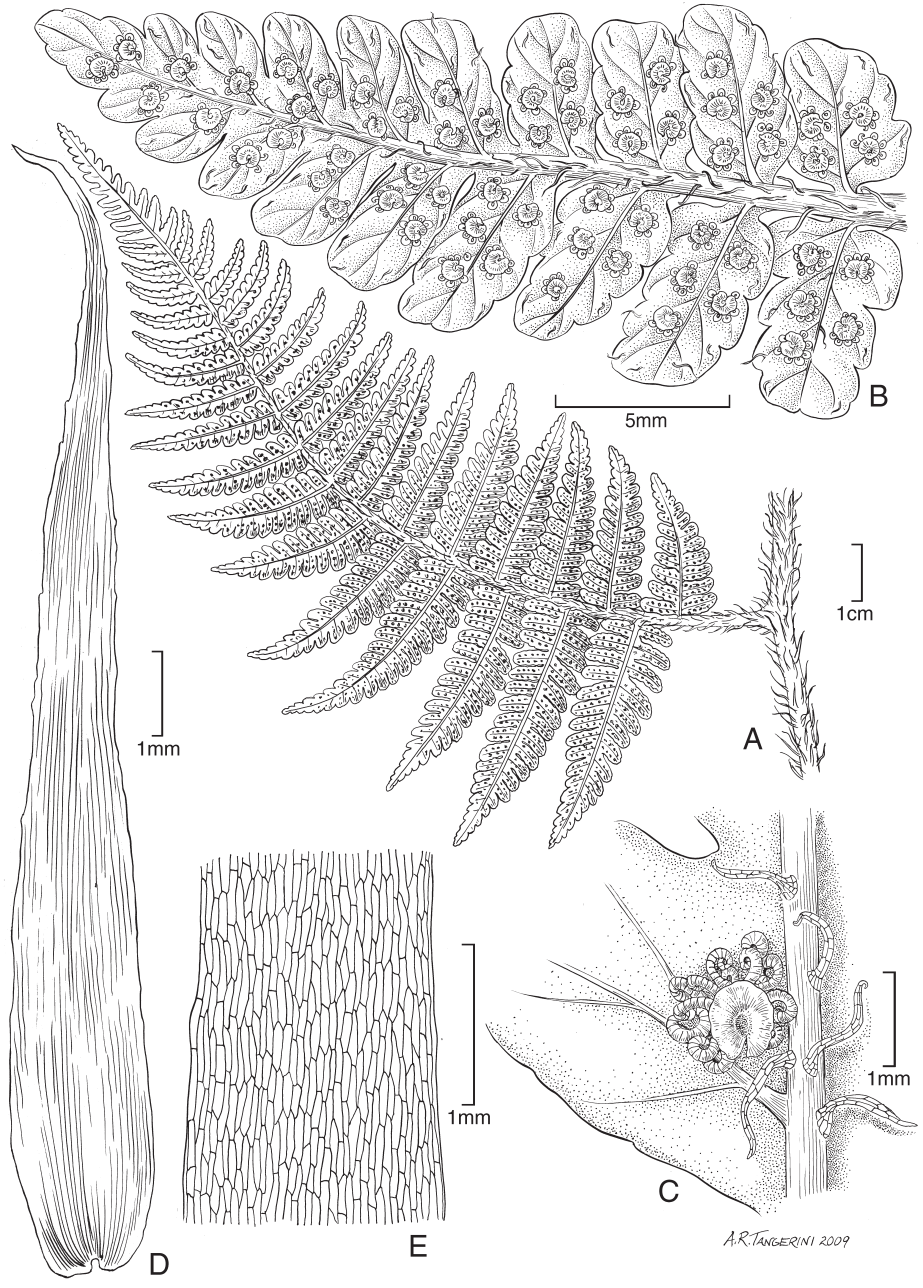
*Dryopteris macropholis* Lorence & W. L. Wagner. **A** pinna from near base of lamina, lower surface **B** lower surface of fertile pinnule showing sori **C** pinnule with sorus, sporangia, and scales **D** scale from stipe **E** part of stipe scale showing detail of cells. Drawn from the type collection (Wood 10489) and field images.

###### Distribution.

Marquesas Islands, known from Nuku Hiva, Ua Huka, Ua Pou, Hiva Oa, and Tahuata.

###### Ecology.

This new species is rare and localized from ca. 700 to 1150 m elevation. It occurs in transitional mesic to wet forests with *Alsophila tahitensis* Brack., *Hernandia nukuhivensis* F. Br., and *Sphaeropteris* spp.; in wet forests dominated by *Crossostylis biflora*, *Freycinetia* spp., *Hibiscus tiliaceus* L., *Metrosideros collina*, *Pandanus tectorius*, and with associates including *Fagraea berteroana* A. Gray ex Benth.*, Ficus prolixa* G. Forst. var. *prolixa*; *Glochidion (Phyllanthus) marchionicum* F. Br.*, Weinmannia marquesana* F. Br. var. *marquesana*, and *Xylosma suaveolens* (J. R. Forst. & G. Forst.) G. Forst. subsp. *pubigerum* Sleumer;in montane wet forests of *Metrosideros collina* and *Weinmannia marquesana* var. *marquesana*; in montane shrublands;and in and summit cloud forests and shrublands with *Alsophila tahitensis*, *Cyrtandra* spp., *Dicranopteris linearis*, *Freycinetia* spp.*, Metrosideros collina*, *Psychotria* spp., *Sphaeropteris* spp., and *Vaccinium cereum* (L. f.) G. Forst. var. *adenandrum* (Decne.) F. Br.and diverse pteridophytes. Threats in most areas include human disturbance, feral pigs, and invasive weeds.

###### Conservation status.

Proposed IUCN Red List Category **Endangered** (EN): B2a, B2b (i–iii): B2: total area of occupancy less than 5000 km2 (ca. 904 km2). B1a, severely fragmented; B1b (i–iii), habitat continuing decline inferred. The suitable habitat for *Dryopteris macropholis* on Nuku Hiva (ca. 340 km2), Ua Huka (ca. 83 km2), Ua Pou (ca. 105 km2), Hiva Oa (ca. 315 km2), and Tahuata (ca. 61 km2) is restricted to mountain slopes and summits, indicated as an endangered environment that is threatened by human activity (deforestation and fire), feral animals, and invasive plants, reducing the extent of the forest.

###### Specimens examined.

**Marquesas Islands: Nuku Hiva.** Toovii, Ooumu area, top of Tapueahu Valley off new road, 8°51'S, 140°19'W, 3500–3700 ft (1067–1128 m), Wood & Perlman 4606 (P, PTBG [2 sheets], US). Route Taiohae–Toovii, branche droite de la haute Taipivai, 8°53'S, 140°8'W, 750 m, Florence 8423 (P [6 sheets]); Piste Nord de Terre Déserte, haute vallée de Tapueahu, 1150 m, 140°11'W, 8°52'S, Florence et al. 9447 (P [3 sheets]). **Ua Huka:** Hitikau and the Vaikivi summit region, 8°54'S, 139°31'W, 800 m, Wood & Perlman 10761 (PAP, PTBG, US). **Ua Pou:** forested ridges and slopes to the N and W of Pouakei, 9°23'S, 140°5'W, 2300 ft (701 m), Wood & Perlman 10840 (PAP, PTBG, US); Tekohepu, 2500–3000 ft (762–914 m), 9°24.31'S, 140°4.21'W, Wood 6499 (P [2 sheets], PTBG [2 sheets], US). **Hiva Oa**: Temetiu, 9°49'S, 139°4'W, 3700 ft (1128 m), Wood 4381 (P, PTBG [2 sheets]); Temetiu region, drainages to southeast of Vaimete et Vaiumioi (source), headwaters of Hanamenu, UTM 0710665–8916125, 3500 ft (1067 m), Wood 10045 (PTBG [5 sheets]); piste de Hanamenu, NW du Mt. Temetiu, 1150 m, 9°48'S, 139°5'W, Florence & Perlman 9665 (P [4 sheets]). **Tahuata:** summit ridge near Haaiputeomo, NE of Vaitahu, 9°57.19'S, 139°5.74'W, 2500–2700 ft (762–823 m), Wood 6572 (BISH, P, PAP, PTBG [5 sheets], US).

###### Discussion.

This remarkable new species resembles *Dryopteris macrolepidota* Copel. (type from Tahiti), a species distinguished by it massive suberect rhizome with russet scales to 30 × 3 mm, fronds to 160 cm long, stipes to 50–70 cm long, stipes and rachises paleaceous with long, thin russet scales with dark, thickened bases, 3-pinnate subcoriaceous blades to 80 × 40 cm, the abaxial surfaces with scattered thin, linear scales, and supramedial sori. Society Islands collections in the Bishop Museum herbarium identified as *Dryopteris dicksonioides* (Mett. ex Kuhn) Copel. have massive prostrate to erect rhizomes to 50 cm tall, fronds 80–140 cm long with stipes 60–100 cm long scaly only near the base, stipe scales dark brown and opaque to 20 mm long, rachises with small brown, scattered scales, and 3-pinnate-pinnatifid blades to 80 × 40 cm, the segments thinly chartaceous with linear brown scales on the lower surfaces. Scales of the rhizomes and stipe bases in *Dryopteris macropholis* are much larger than in the former two species. Palmer (2003) considered *Dryopteris dicksonioides* synonymous with *Dryopteris glabra* (Brack.) Kuntze, an exindusiate species endemic to the Hawaiian Islands. However, the type of *Dryopteris dicksonioides* is from Tahiti and clearly does not represent *Dryopteris glabra* (Copeland 1932). This new species seems most closely related to *Dryopteris sweetorum*, known only from Fatu Hiva, which differs by its rhizome and stipe base scales smaller, lustrous, dark brown, 9–25 × 1–2 mm and its broadly ovate-deltate, 3-pinnate-pinnatifid blades (42–)62–93 × (38–)64–80 cm, the ultimate segments to 15 × 5 mm, spaced 5–10 mm distant, oblique with tips acute, cut about halfway to the costule, with margins acutely serrate, and the abaxial surfaces and rachises glabrous.

##### 3. Dryopteris sweetorum

Lorence & W. L. Wagner sp. nov.

urn:lsid:ipni.org:names:77112675-1

http://species-id.net/wiki/Dryopteris_sweetorum

Fig. 5 

###### Latin.

A D. macropholi minoribus, atro-castaneis, ovato-lanceolatis vel lineari-oblongis squamis 12-25 × 1.5-2 mm supra rhizomates et stipitum bases, largis tripinnato-pinnatifidibus frondibus, ultimis segmentibus usque ad 15 × 5 mm, intervallibus 5-10 mm distantibus, obliquis, acutis culminibus, sectis ca. in medio ad costulam cum marginibus acute serratis, abaxialibus superficiebus et glabris rachidibus differt.

###### Type.

**Marquesas Islands: Fatu Hiva:** Teavapuhiau, ridge to Touaouoho, 2000 ft (607 m), 8 September 1995, K. R. Wood 4493(Holotype: PTBG-038471!, PTBG-038472!, PTBG-038473!, PTBG-0384741! [4 sheets]; Isotypes BISH! [2 sheets], P!, PAP! [3 sheets], NY!, US! [3 sheets]).

###### Description.

*Terrestrial ferns*; *rhizomes* short creeping to suberect, 3–15 cm in diameter including stipe bases, densely clothed with scales; scales of rhizomes and bases of stipes lustrous, dark brown, 9–25 × 1–2 mm, ovate-lanceolate to linear-oblong, concolorous, margins entire to subentire distally with short, irregular teeth, cells linear. *Fronds* densely clustered, 5–7 per rhizome; stipes (45–)60–100 cm long, 7–10 mm in diameter medially, adaxially grooved, stramineous to medium brown, densely scaly basally, distally with progressively smaller scales and appressed hairlike scales, mostly glabrescent, surfaces brown-punctuate; blades firmly chartaceous, broadly ovate-deltate, (42–)62–93 × (38–)64–80 cm, 3-pinnate-pinnatifid at base, otherwise mostly 2-pinnate-pinnatifid, with pinnae opposite to subopposite, spreading, 8–13 on a side; rachises stramineous, with scattered narrow scales to 2–12 × 0.2–0.6 mm; lowermost pinnae the largest, 25–38 × 19–25 cm, slightly inequilateral, basiscopic basal pinnules 10–15 cm long, acroscopic basal pinnules slightly shorter, 7–12 cm long, distal pinnae stalked to 5 mm or sessile, apex pinnatifid; ultimate pinnules to 15 × 5 mm, spaced 5–10 mm distant, oblique, tips acute, cut ca. ½ toward costule, with margins acutely serrate, adaxially glabrous, abaxially glabrous or rachises with scattered hairlike scales; veins forking, scarcely visible adaxially, prominulous abaxially. *Sori* (1–)3–6 pairs per segment, supramedial; indusia 0.9–1.3 mm in diameter, brown, thick, glabrous except with a few short-stipitate glands in center. *Spores* dark brown.

**Figure 5.**
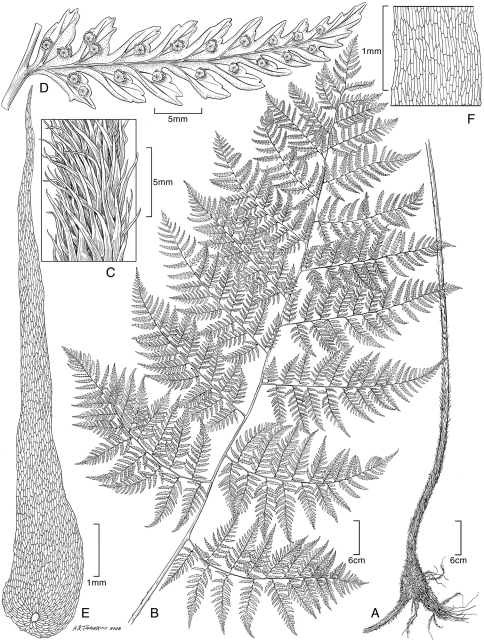
*Dryopteris sweetorum* Lorence & W. L. Wagner. **A** stipe and part of rhizome **B** blade **C** detail of stipe with scales **D** lower surface of fertile pinnule showing sori **E** rhizome scale **F** part of rhizome scale, detail of cells. Drawn from the type collection (Wood 4493*)* and field images.

###### Distribution.

Known only from Fatu Hiva, Marquesas Islands, in montane wet forest at 600–640 m elevation.

###### Ecology.

This new endemic species is apparently rare and localized, known only from the region from Teavapuhiau ridge to Mt. Touaouoho where it grows scattered among other ferns on hillsides in understory of open forest with *Alsophila tahitensis*, *Crossostylis biflora*, *Cyclophyllum barbatum* (G. Forst.) N. Hallé & J. Florence, *Freycinetia impavida*, *Hibiscus tiliaceus*, *Metrosideros collina*, *Pandanus* sp.*, Weinmannia marquesana* var. *marquesana*, and *Vaccinium cereum* var. *adenandrum*, and *Wikstroemia coriacea* Seem.

###### Etymology.

We take great pleasure in naming this magnificent new species in honor of Barbara K. and Cyrus B. Sweet, III, who have generously supported scientific research at the National Tropical Botanical Garden and particularly the Vascular Flora of the Marquesas Islands project.

###### Discussion.

This new species differs from *Dryopteris macropholis* by the characters noted in the diagnosis above, notably in the ultimate pinnules segments spaced 5–10 mm distant with serrate margins and acute apices.

###### Conservation status.

Proposed IUCN Red List Category: **Critically Endangered** (CR): B1ab, B2a,b (i–iii). B1, total extent of occurrence less than 100 km2 (ca. 85 km2), a,b, known from a single location; B2a, estimated area of occupancy estimated to be less than 10 km2 [three collections likely representing a single population are known]; B2b (i–iii), habitat continuing decline inferred. The estimated area of occupancy for *Dryopteris sweetorum* on Fatu Hiva (less than 10 km2) is indicated as an endangered environment, threatened by human activity (deforestation and fire), feral animals, and invasive plant species, reducing the extent of the forest.

###### Specimens examined.

**Marquesas Islands:** Fatu Hiva: Teavapuhiau, leeward side of windswept ridge, UTM 758470–8840707, 2100 ft (640 m), Wood 10091 (PTBG [3 sheets], PAP [2 sheets], US [3 sheets]); Teavapuhiau Pass (above Ouia Valley), 700 m, B. Gagné 1233 (US); Epaulement SW de Mt. Touaouoho, 770 m, 10°29'S, 138°38'W, Florence et al. 9536(P [4 sheets]).

#### Polystichum

*Polystichum* Roth is a cosmopolitan genus of about 300 species (Mabberley 2008). Brown and Brown (1931) recognized and described a single species from the Marquesas, *Polystichum marquesense* E. D. Br. Recent collections from Hiva Oa, Tahuata, and Ua Huka have revealed the presence of two new species which are described below. *Polystichum marquesense* differs from both new species in having exindusiate sori and stipes clothed with large, dull brown, ovate, overlapping scales.

Key to *Polystichum* in the Marquesas Islands

**Table:** 

1a	Blades uniformly bipinnate	*Polystichum uahukaense*
1b	Blades tripinnate, at least in lower portion	2
2a	Largest stipe scales linear-oblong, 25–30 × 1–3.5 mm, reddish brown with margins bearing short acicular teeth, concentrated base of stipe, mid to upper part of stipe with smaller, spreading linear-oblong scales and hairlike scales; largest pinnules and lobes of largest pinnae usually with 1 apical and occasionally 1–6 crenate marginal teeth, arista of apical tooth 0.5–0.7 mm long; indusia present	*Polystichum kenwoodii*
2b	Largest stipe scales ovate to narrowly ovate, 10–20(–30) × 4–10 mm, dull, pale brown with strongly erose-ciliate margins, overlapping and evenly distributed along stipe, mixed with smaller ovate-ciliate scales and cobwebby scurf; largest pinnules and pinnule lobes of largest pinnae usually with 3–8(–11) teeth, arista of apical tooth 0.5–2 mm long; indusia absent	*Polystichum marquesense*

##### 4. Polystichum kenwoodii

Lorence & W. L. Wagner sp. nov.

urn:lsid:ipni.org:names:77112677-1

http://species-id.net/wiki/Polystichum_kenwoodii

Figs 6 14E 

###### Latin.

A Polytsicho marquisensi E. D. Br. longioribus et angustis rhizomatibus, stipitis squamis 20-35 mm, squamis prope stipitis basin congregates, soris indusiatis differt.

###### Type.

**Marquesas Islands:** Ua Huka: Vaikivi summit region and drainage, 8°54'S, 139°31'W, 600 m, 16 June 2004, K. R. Wood 10759 (Holotype PTBG-044161!, PTBG-044162!, PTBG-044163! [3 sheets]; Isotypes BISH!, P!, PAP!, US!).

###### Description.

*Terrestrial ferns*; *rhizomes* erect or suberect, 7–20 cm long, 5–15 cm in diameter including bases of stipes (from collector’s notes); rhizome scales 20–35 × 2–2.5 mm, linear-oblong, concolorous, medium brown, margins entire, cells narrow linear, in sinuous files, mixed with slender hairlike scales. *Fronds* clustered; stipes 55–90 cm long, 5–6 mm in diameter medially, 2/5 as long as or equaling length of the blade, adaxially grooved, pale brown, basally densely clothed with reddish brown, linear to linear-oblong scales 10–30 × 1.5–3 mm, similar to rhizome scales, stipes distally with progressively smaller and finer scales mixed with matted hairlike scales or glabrescent; *blades* narrowly ovate to oblong-ovate, 50–83 × 30–32 cm, tripinnate at least in lower pinnae, with 20–28 pinnae on a side; rachises stramineous, moderately to densely scaly with reddish brown hairlike scales to 8 mm long; pinnae linear-oblong, apices long-acuminate, largest pinnae 8–26 × 4.5–7 cm, lowermost2(–3) pinnae pairs slightly reduced, costules adaxially grooved; largest pinnae with up to 32 pairs of pinnules, pinnules deeply incised acro- and basiscopically forming 1–2 free, obovate lobes or blades tripinnate in large fronds, pinnule apices obtuse, tips abruptly acuminate-aristate, both margins serrate-crenate, blades stiffly chartaceous to subcoriaceous, adaxially glabrous except for a few hairlike scales basally, abaxially with whitish or tan hairlike scales with expanded lacerate-ciliate bases on costules and veins; veins forking, scarcely visible adaxially, slightly prominulous abaxially. *Sori* with a peltate *indusium* 0.5–0.7 mm in diameter, tan, thin and fugacious, with scattered marginal projections, not confluent at maturity. *Spores* dark brown.

**Figure 6.**
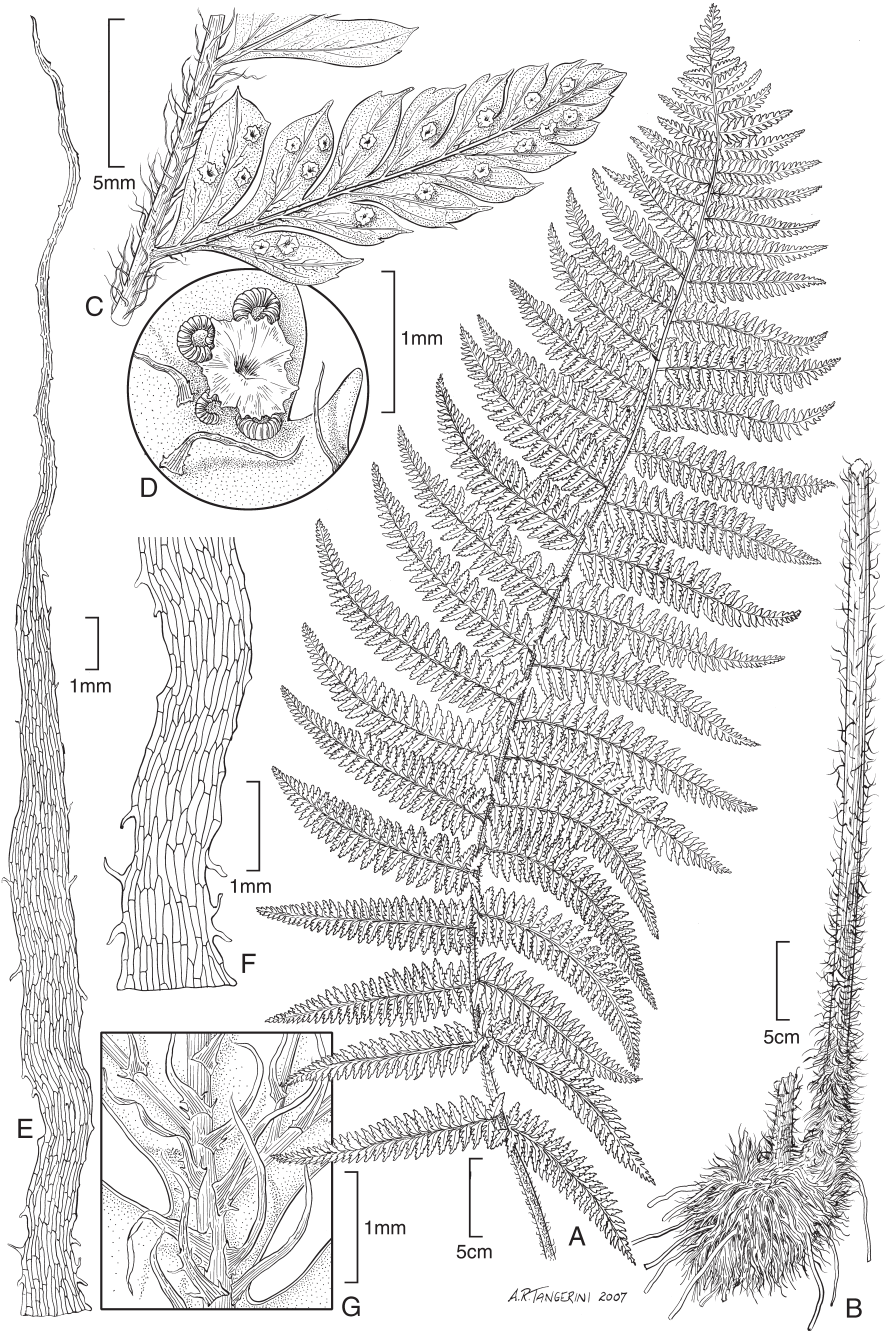
*Polystichum kenwoodii* Lorence & W. L. Wagner. **A** blade **B** stipe and part of rhizome **C** lower surface of fertile pinnule showing sori and scales **D** sorus with indusium and scales **E** rhizome scale **F** part of rhizome scale, detail of cells **G** lower surface of sterile pinnule showing scales. Drawn from type collection (Wood 10759) and field images.

###### Distribution.

Marquesas Islands, known from Ua Huka, Hiva Oa, and Tahuata.

###### Habitat.

This new species occurs at 600–884 m elevation in diverse montane wet forest and shrubland dominated by *Freycinetia* sp.*, Hibiscus tiliaceus*, and *Weinmannia marquesana* var. *marquesana* with other associates including *Alstonia marquisensis* M. L.Grant*, Boehmeria virgata* (G. Forst.) Guill.*, Cheirondendron bastardianum* (Decne.) Frodin*, Crossostylis biflora*, *Cyclophyllum barbatum*, *Ficus prolixa* var. *prolixa, Metrosideros collina, Myrsine grantii* Fosberg & Sachet, *Reynoldsia marchionensis* F. Br.*, Xyolosma suaveolens* subsp. *pubigera*, and often growing with numerous other pteridophytes.It also occurs in transitional mesic to wet forest with *Alsophila tahitensis*, *Crossostylis biflora*, *Freycinetia* sp.*, Metrosideros collina*, *Pandanus tectorius*, and *Weinmannia marquesana* var. *marquesana*. *Polystichum kenwoodii* grows terrestrially in forest understory or sometimes along boulder-strewn stream beds.

###### Etymology.

We take pleasure in naming this new species for its collector Kenneth R. Wood (1953–), whose excellent collections have contributed greatly to our knowledge of the Marquesas flora.

###### Conservation status.

Proposed IUCN Red List Category: **Endangered** (EN): B2a, B2b (i–iii): B2: total area of occupancy less than 500 km2 (ca. 460 km2); B2a, severely fragmented with only three populations known; b (i–iii), habitat continuing decline inferred. The suitable habitat for *Polystichum kenwoodii* on Ua Huka (ca. 83 km2), Hiva Oa (ca. 315 km2), and Tahuata (ca. 61 km2) is indicated as an endangered environment, threatened by human activity (deforestation and fire), feral animals, and invasive plants, reducing the extent of the forest.

###### Specimens examined.

**Marquesas Islands: Hiva Oa:** Hanamenu region, up Hanamenu valley to the drainages below and west of Temetiu, 9°76'S, 139°0'W, 884 m, K. R. Wood 10232 (BISH, P, PAP, PTBG [3 sheets], US). **Tahuata:** Amatea region, around Haaoiputeomo satellite dish, 9°92'S, 139°8'W, 884 m, K. R. Wood 10257(BISH, P, PAP, PTBG [3 sheets], US).

###### Discussion.

*Polystichum rapense* E. D. Br. from Rapa Iti (Austral Islands) resembles this new species but differs in having very dark brown, almost black scales 3–4 mm wide on the rhizome and base of stipe and much smaller, bipinnate fronds (stipes 6–10 cm long, blades 11–14 × 8–10 cm). *Polystichum stokesii*, also from Rapa Iti, has similar dark brown to blackish, lustrous scales on the rhizome and base of stipe and shorter fronds with stipes to 20 cm long and blades to about 50 × 40 cm compared with *Polystichum kenwoodii*.

##### 5. Polystichum uahukaense

Lorence & W. L. Wagner sp. nov.

urn:lsid:ipni.org:names:77112678-1

http://species-id.net/wiki/Polystichum_uahukaense

Figs 7 14F, 15A 

###### Latin.

A Polytsicho marquisensi E. D. Br. minoribus rhizomatibus 3-4 cm. longis × 1.5-3 cm diametro, frondibus minoribus bipinnatibus cum laminis 26-58 × 12-27 cm, soris indusiatis differt.

###### Type.

**Marquesas Islands: Ua Huka**: Hane/Hokatu cliff zone, 520 m, 11 December 2003, K. R. Wood & J.-Y. Meyer 10518 (Holotype PTBG-042930!; Isotypes AD!, BISH!, K!, MO!, NY!, P!, PAP!, UC!, US!).

###### Description.

*Terrestrial ferns*; *rhizomes* short, erect or suberect, 3–4 cm long, 1.5–3 cm in diameter; rhizome scales 20–28 × 2–2.5 mm, linear-oblong, concolorous, medium to dark brown, margins entire or with occasional short teeth, cells linear, in sinuous files. *Fronds* 5–7 per rhizome; stipes 16–43 cm long, ½–2/3 length of the blades, adaxially grooved, base with dense, linear to linear-oblong scales 10–20 × 1–2 mm, brown and similar to rhizome scales but with thinner, lacerate-dentate margins, mixed with smaller hairlike scales with lacerate bases, mid- to upper part of stipe with hairlike scales or glabrescent; blades ovate-oblong to narrowly oblong, 26–58 × 12-27 cm, bipinnate; rachises densely scaly with pale brown, hairlike scales 3-6 mm long with expanded lacerate base; pinnae 20–30 on a side, narrowly oblong, apex narrowly acute, largest pinnae 6–15 × 1.5–3 cm, lowermost 1(–2) pairs slightly reduced, costules adaxially grooved, pinnules 8–16 pairs per pinna, each incised acroscopically with a small auricle or basal pinnules of larger pinnae sometimes deeply incised forming a nearly free obovate lobe, pinnule apices acute to obtuse, both margins crenate, apex and lobes spinulose at tip; blades chartaceous to subcoriaceous, adaxially sparsely capitate-glandular when young, glabrate, abaxially with pale tan hairlike scales on costae, veins, and margins, veins forking, scarcely visible adaxially, slightly prominulous abaxially. *Sori* each with a peltate indusium 0.5–0.6 mm in diameter, tan, with numerous marginal projections, thin and often fugacious, not confluent at maturity. *Spores* black.

**Figure 7.**
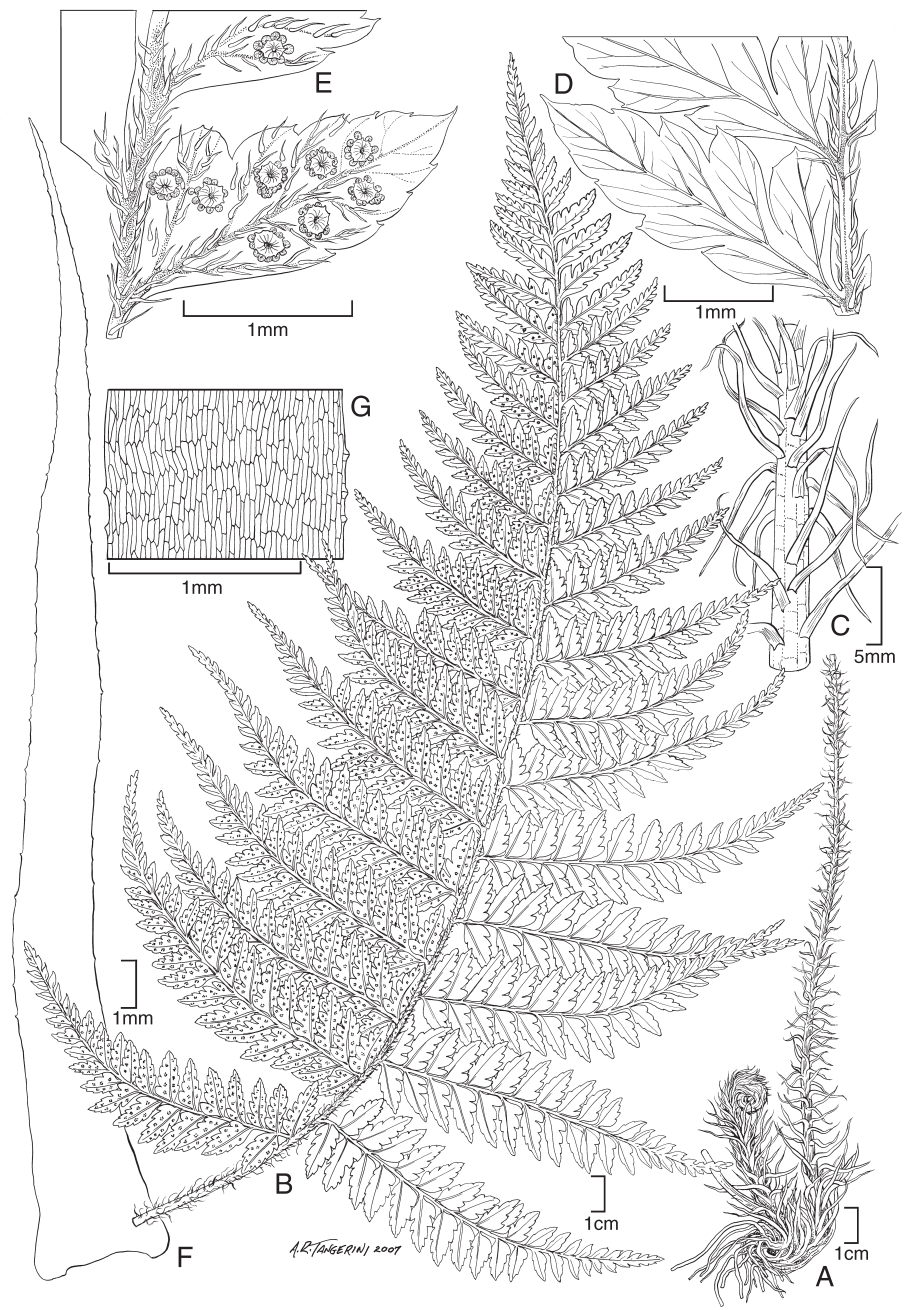
*Polystichum uahukaense* Lorence & W. L. Wagner. **A** rhizome and stipe **B** blade **C** detail of stipe with scales **D** lower surface of sterile pinnule **E** lower surface of fertile pinnule showing sori and scales **F** rhizome scale, outline **G** part of rhizome scale, detail of cells. Drawn from the type collection (Wood 10518) and field images.

###### Distribution.

Marquesas Islands, known only from the type locality on Ua Huka in the Hane/Hokatu cliff zone at the head of the valley above Hane village.

###### Ecology.

*Polystichum uahukaense* is extremely localized and known from a single population on moist, mossy cliff faces in shade of wet forest with *Hibiscus tiliaceus* and *Pandanus tectorius* dominant, at 520–525 m. Associated vegetation on cliffs includes *Bidens polycephala* Sch. Bip., *Boehmeria virgata*, *Macropiper latifolium* (L. f.) Miq., *Peperomia pallida* (G. Forst.) A. Dietr., *Phyllanthus pacificus* Müll. Arg., and pteridophyes including *Dryopters macropholis*, *Microsorum grossum* (Langsd. & Fisch.) Brownlie*, Nephrolepis* sp., *Pteris comans* G. Forst., *Selaginella arbuscula* (Kaulf.) Spring., and *Tectaria jardinii* (Mett. ex Kuhn) E. D. Br. The main threats to this ecosystem include rockslides and competition with naturalized plant species including *Ageratum conyzoides* L.*, Coffea arabica* L.*, Kyllinga brevifolia* Rottb., *Oplismenus compositus* (L.) P. Beauv., *Paspalum conjugatum* P. J. Bergius*, Psidium guajava* L., and *Zingiber zerumbet* (L.) Sm.

###### Etymology.

This new species is named for its only known island of occurrence.

###### Conservation status.

Proposed IUCN Red List Category **Critically Endangered** (CR): B2a, B2b (i–iii); D): B2: total area of occupancy less than 10 km2 (ca. 5 km2). B2a, a single population known; b (i–iii), habitat continuing decline inferred. The suitable habitat for *Polystichum uahukaense* on Ua Huka (ca. 83 km2) is indicated as an endangered environment, threatened by feral animals and invasive plants, reducing the extent of the forest. Estimated population size is 300 individuals (*Wood 10735*).

###### Specimens examined.

**Marquesas** **Islands: Ua Huka:** Hane/Hokatu cliff zone, 520 m, 14 December 2003, K. R. Wood & J.-Y. Meyer 10552 (P, PAP, PTBG, US), 480 m, 8°54'37"S, 139°31'32"W, 12 June 2004, Lorence et al. 9303 (BISH, NY, P, PAP, PTBG, US).

###### Discussion.

*Polystichum uahukaense* differs from its Marquesas Islands congeners in having uniformly bipinnate blades, a feature it shares with *Polystichum rapense* from Rapa Iti which differs in having very dark brown, almost black scales 3–4 mm wide on the rhizome and base of stipe and much smaller, bipinnate fronds (stipes 6–1 cm long, blades 11–14 × 8–10 cm). However, based on overall morphology these two species seem closely allied to each other.

### PTERIDACEAE

#### Pteris

*Pteris* L. is a large, pantropical genus comprising about 250 species (Mickel and Smith 2004). In their treatment of southeastern Polynesian pteridophytes, Brown and Brown (1931) recognized two indigenous species of *Pteris* L. from the Marquesas: *Polystichum comans* G. Forst. and *Polystichum tripartita* Sw. Recent collections made by Kenneth R. Wood in conjunction with this project have yielded three additional, very distinctive endemic *Pteris* species described below.

Key to *Pteris* in the Marquesas Islands

**Table:** 

1a	Blade tripartite	*Polystichum tripartita*
1b	Blade 1- to 2-pinnate	2
2a	Blade 2-pinnate	*Polystichum comans*
2b	Blade 1-pinnate to 1-pinnate-pinnatifid	3
3a	Stipes thickly clothed in persistent, stiff, spreading, red-brown bristlelike or hairlike scales	*Polystichum marquesensis*
3b	Stipes glabrous or soon glabrate	4
4a	Apex pinnatifid, 6–7-lobed	*Polystichum hivaoaensis*
4b	Apex entire, the single terminal pinna free or sometimes adnate basally to the distal pinnae pair	*Polystichum tahuataensis*

##### 6. Pteris hivaoaensis

Lorence & K. R. Wood sp. nov.

urn:lsid:ipni.org:names:77112679-1

http://species-id.net/wiki/Pteris_hivaoaensis

Figs 8 15B 

###### Latin.

Ab aliis Marquesas speciebus laminis 1-pinnatis usque ad 1-pinnato-pinnatifidis, apice cum 6-7 loborum paribus distalibus falcatis, glabris vel glabrescentibus stipitibus differt.

###### Type.

**Marquesas Islands:** Hiva Oa: Temetiu, windswept ridges and drainages, 9°49'S, 139°4'W, 930 m (3050 ft), 24 August 1995, K. R. Wood 4374(Holotype PTBG-038496!; Isotypes BISH!, P!, PAP!, US!).

###### Description.

*Lithophytic ferns*; *rhizomes* erect, clumping together, 3–7 cm long,15–22 mm in diameter, clothed in very narrow, golden-brown acicular hairlike scales 1–2 mm long. *Fronds* erect or arching-pendent, to 30 cm long, clustered at rhizome apex; *stipes* ca. ½ length of frond, up to 1 mm in diameter medially, atrocastaneous, grooved adaxially, sparsely pustulate-tuberculate, glabrous except for a few linear to linear-lanceolate, falcate scales 2–3 × 0.1–0.5 mm at bases, tan-brown, margins subentire; *blades* chartaceous, glabrous, narrowly ovate to lanceolate, 10–16 × 5–9 cm; proximal 2–3 pinnae consisting of subopposite, subequal pairs, 1-pinnate-pinnatifid for most of length, sessile or stalked up to 2 mm, non-articulate, bearing 2–3 pair of falcate lobes of increasing size proximally with maximum size at basiscopic lobe of lowest pinnae, lobes linear-oblong or linear-triangular 0.3–2.5 × 0.3–11 mm, apices rounded to acute, crenate to dentate, basal pinnae slightly reduced, blade distally pinnatifid, lanceolate, the apex pinnatifid, with 6–7 pairs of falcate lobes 5–8 mm wide above their bases, dilated basiscopically, margins mostly entire, tapering gradually to acute apices with crenate-dentate margins, single terminal lobe falcate or tapering to narrowly linear crenate margin; margins of sterile pinnae finely dentate with one tooth per vein ending; costae and costules grooved adaxially, rounded abaxially, similar to stipe in color; veins netted with 1–2 rows of areoles. *Sori* occasionally interrupted at sinuses and absent at apices of segment; indusia tan-brown with entire margins. *Spores* tan-brown.

**Figure 8.**
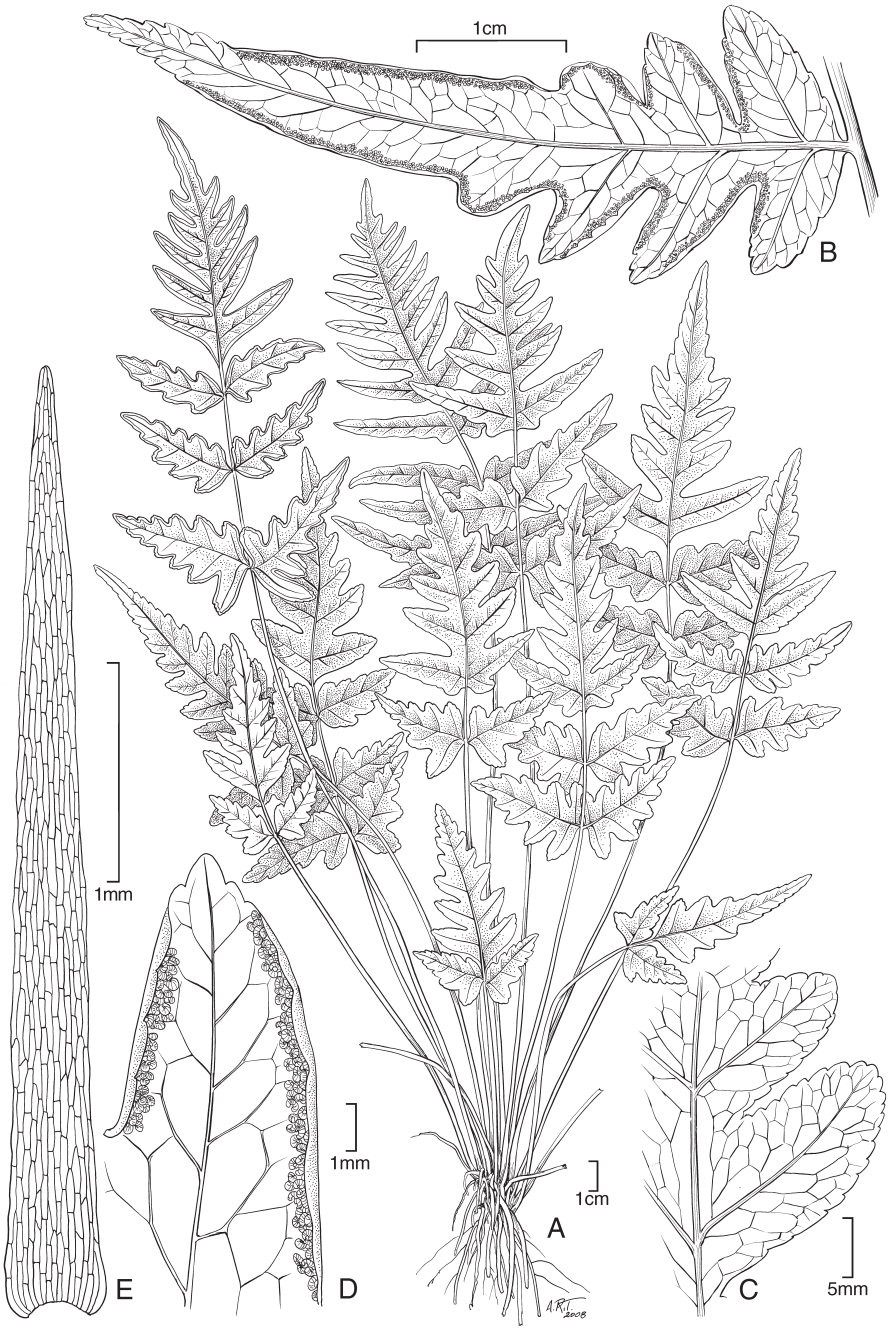
*Pteris hivaoaensis* Lorence & K. R. Wood. **A** habit **B** lower surface of fertile pinna **C** sterile pinnule, detail of venation **D** lower surface of fertile pinnule showing sori **E** rhizome scale. Drawn from the type collection (Wood 4374) and field images.

###### Distribution.

Known only from Hiva Oa, Marquesas Islands.

###### Ecology.

This rare species is known only from the type location at 930 m on wet stream walls adjacent to wet forest dominated by *Cheirodendron bastardianum*, *Crossostylis biflora*, *Metrosideros collina*, * Weinmannia marquesana* var. *marquesana* and other associates including *Alsophila tahitensis*, *Cyrtandra* spp., *Freycinetia* sp., *Leptochloa marquisensis* (F. Br.) P. M. Peterson & Judz., *Melicope* spp., and *Psychotria* spp., interspersed with a rich pteridophyte understory. The main threats to this habitat are feral pigs and invasives plants including *Elephantopus mollis* Kunth, *Psidium guajava*, and *Syzygium cumini* (L.) Skeels.

###### Conservation status.

Proposed IUCN Red List Category **Critically Endangered** (CR): B2a, B2b (i–iii), D); B2: total area of occupancy less than 10 km2 (ca. 5 km2). B2a, a single population known; b (i–iii), habitat continuing decline inferred; D, population estimated to number fewer than 250 mature individuals. The suitable habitat for *Pteris hivaoaensis* on Hiva Oa (ca. 315 km2) is indicated as an endangered environment, threatened by human activity (deforestation, fire), feral animals, and invasive plants, reducing the extent of the forest. D, the rarity of this species is supported by the lack of collections and the small extant area within a commonly collected island, i.e. with a single collection.

###### Etymology.

This new species is named for its only known island of occurrence.

###### Discussion.

*Pteris hivaoaensis* is quite distinct from the other Marquesan *Pteris* species, differing by its pinnate to pinnate-pinnatifid blades with glabrous or glabrescent stipes and the apex pinnatifid with 6–7 pairs of falcate lobes.

##### 7. Pteris marquesensis

Lorence & K. R. Wood sp. nov.

urn:lsid:ipni.org:names:77112680-1

http://species-id.net/wiki/Pteris_marquesensis

Figs 9 15C, D. 

###### Latin.

Ab aliis Marquesas speciebus laminis 1-pinnatis usque ad 1-pinnato-pinnatifidis, stipitibus dense vestitis cum squamis persistentibus, rigidibus, setiformibus rubro-castaneis differt.

###### Type.

**Marquesas Islands:** Tahuata, summit ridge near Haaiputeomo, satellite dish region NE of Vaitahu, 2500–2700 ft, 9°57.19'S, 139°5.74'W, 17–19 July 1997, K. R. Wood 6565(Holotype PTBG 038520!, 038521!, 038522!, 038523!, 038524! [5 sheets]; Isotypes BISH!, NY!, P!, PAP!, UC!, US!).

###### Description.

*Terrestrial ferns*; *rhizomes* dorsiventral, short creeping to suberect, up to 2 cm in diameter (to 15 × 15 cm including stipe bases), scaly at apex, scales of rhizome and base of stipe concolorous, reddish brown, lustrous, linear, sinuate distally, 15–30 × 0.5–0.7 mm, margins subentire or rarely with scattered short teeth, intermixed with smaller narrower scales. *Fronds* 4–7 per rhizome, clustered at rhizome apex; stipes 60–134 cm long, 4–6 mm in diameter medially, adaxially grooved, stramineous, densely scaly especially in lower ca. 1/3, above with scales spreading and less dense, acicular, bases dark brown, persistent, thickened, distally sinuate, apex filiform, margins subentire; lamina chartaceous, 1-pinnate-pinnatifid or in smaller fronds 1-pinnate, 66–90 × 35–64 cm, ovate-triangular, base obtuse or truncate, apex acute, pinnatifid and not conform; pinnae 6–10 pairs, basal 1–5 pinnae pairs rarely entire or more commonly irregularly pinnatifid or lobed at least toward base, or occasionally to apex in sterile fronds, basal pair of pinnae on stalks 2–6 mm long, largest basal pinnae 22–36 × 11–18 cm, basiscopic lobes to 13 × 2.5 cm, linear-oblong, slightly falcate, nearly twice as long as acroscopic lobes, acroscopic base often auriculate, apex acute to acuminate, occasionally obtuse in shorter lobes, middle and upper pinnae sessile, basiscopic base decurrent on rachis, acroscopic base free and nearly parallel to rachis, margins entire or finely serrate towards lobe apices, rachis stramineous, grooved adaxially, with scattered dark brown, acicular scales to 2 mm long, glabrescent or scales sometimes persisting in pinnae axes; *veins* netted with 2–5 rows of areoles in larger pinnules. *Sori* with indusia 1 mm wide, entire, continuous along margins except at serrate apices; sporangia mixed with paraphyses. *Spores* medium brown.

**Figure 9.**
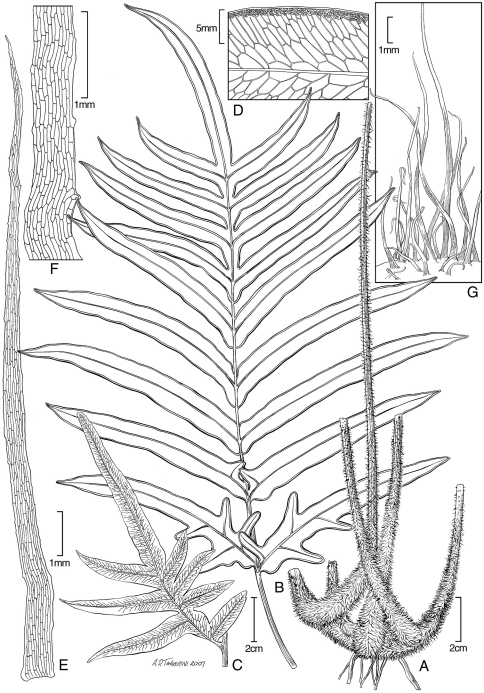
*Pteris marquesensis* Lorence & K. R. Wood. **A** rhizome and stipe bases **B** fertile blade **C** lower sterile pinna **D** lower surface of fertile pinnule showing marginal sori **E** rhizome scale **F** part of rhizome scale, detail of cells **G** detail of stipe scales. Drawn from the type collection (Wood 6565) and field images.

###### Distribution.

Known from Tahuata and Hiva Oa, Marquesas Islands.

###### Ecology.

*Pteris marquesensis* occurs in wet forest and shrubland with *Crossostylis biflora*, *Freycinetia* sp., *Metrosideros collina*, and *Weinmannia marquesana* var. *marquesana*, dominant. Other associates include *Cyrtandra* spp., *Morinda citrifolia*, *Psychotria* spp.*, Vaccinium cereum* var. *adenandrum*, numerous pteridophytes including *Asplenium* spp.*, Doodia marquesensis* E. D. Br., and *Tmesipteris gracilis* Chinnock, and diverse bryophytes.

###### Etymology.

This new species is named for the Marquesas Islands, where it is known from three islands.

###### Conservation status.

Proposed IUCN Red List Category **Endangered** (EN): B2a, B2b (i–iii), D): B2: total area of occupancy less than 500 km2 (ca. 10 km2); b (i–iii), habitat continuing decline inferred; D, population size estimated to number fewer than 250 mature individuals. The suitable habitat for *Pteris marquesensis* on Hiva Oa (ca. 315 km2) and Tahuata (ca. 61 km2) is indicatedas an endangered environment, threatened by human activity (deforestation, fire), feral animals, and invasive plants, reducing the extent of the forest.

###### Discussion.

This striking, large new species is distinctive among all other Polynesian and Micronesian members of the genus. It resembles *Pteris warburgii* Christ from Papua New Guinea which also has reticulate veins and pinnatifid blades, but that species differs in having only a single pair of lateral pinnae and a terminal pinna, and very few, inconspicuous rhizome and stipe scales. *Pteris marquesensis* also somewhat resembles *Polystichum umbrosa* R. Br. from Australia, but that species has fronds with free dichotomous veins and narrower pinnae adnate to the winged rachis, with the lowermost pair of pinnae pinnate-pinnatifid.

###### Specimens examined.

**Marquesas Islands: Hiva Oa:** Temetiu region, drainages to the SE of Vaimete and Vaiumioi, headwaters of Hanamenu, UTM 0710665–8916125, 1067 m, Wood 10046 (P, PAP, PTBG, US); Ootua, W side of summit area above road, 9°46'S, 138°58'W, 777 m, Wood 10871 (PTBG, 3 sheets), Wood 10072 (P, PAP, PTBG, US). **Tahuata:** Haaoiputeomo near satellite dish, NE from Vaitahu to summit ridge, 2000–2500ft (610–762 m), Wood 4461 (BISH, NY, P, PAP, PTBG, UC, US); satellite dish region NE of Vaitahu, 2500–2700 ft (762–823 m), 9°57.19'S, 139°5.74'W, Wood 6556 (BISH, NY, P, PAP, PTBG, US).

##### 8. Pteris tahuataensis

Lorence & K. R. Wood sp. nov.

urn:lsid:ipni.org:names:77112681-1

http://species-id.net/wiki/Pteris_tahuataensis

Figs 10 15E 

###### Latin.

Ab aliis Marquesas speciebus laminis 1-pinnatis usque ad 1-pinnato-pinnatifidis, distalibus pinnis non alatis, et glabris ver glabrescentibus stipitibus differt.

###### Type.

**Marquesas Islands:** Tahuata: Hanatetena, main valley, first deep gulch to the north, general UTM 0710938 – 8899189, 518 m, 2 Feb 2003,K. R. Wood 10083 (Holotype: PTBG-04297!; Isotypes P!, PAP!, UC!, US!).

###### Description.

*Terrestrial or lithophytic ferns*; *rhizomes* creeping to suberect, 2–5 cm long, 1–3 cm in diameter, clothed in fine, golden-brown acicular hairs 1–2.5 mm long; *fronds* 15-20 per rhizome, pendent, 35–125 cm long; stipes ca. ½ length of frond, up to 4 mm in diameter, atrocastaneous to stramineous, grooved adaxially, sparsely pustulate, glabrous except for a sparse cover of linear to linear-lanceoate, tan-brown scales 3–5 × 0.3–0.5 mm at base of stipes; blades chartaceous, glabrous, ovate, 18–65 × 12–35 cm, ovate. with 5–7 pairs of pinnae; proximal 1–3 pinnaepairs 1–(2–) pinnate-pinnatifid, up to 34 cm long, sessile or stalked up to 9 mm, base obtuse to truncate, uniauriculate or bearing 2–3 pair of falcate lobes or with proximal pair occasionally dividing into falcate pinnules 5–13 cm × 1.4–1.7 mm, reaching maximum length basiscopically on lowest pinnae, apices acute to cuneate, or attenuate, margins crenate to dentate; distal part of blade composed of 3–5 pinnae pairs, these simple, falcate, subopposite, up to 23 cm long, the bases sessile, obtuse to truncate, sometimes uniauriculate acroscopically, tapering gradually to cuneate or attenuate apices with crenate-dentate margins, the single terminal pinna free or sometimes adnate basally to the distal pinnae pair, 6.5–23 × 1.0–2.5 cm, apex attenuate, crenate; costaeand costules grooved adaxially, rounded abaxially, similar to stipe in color; veins netted with 2–3 rows of areoles. *Sori* with indusia 0.5–0.9 mm wide, olivaceous; sori usually absent at apices of pinnae, *Spores* castaneous.

**Figure 10.**
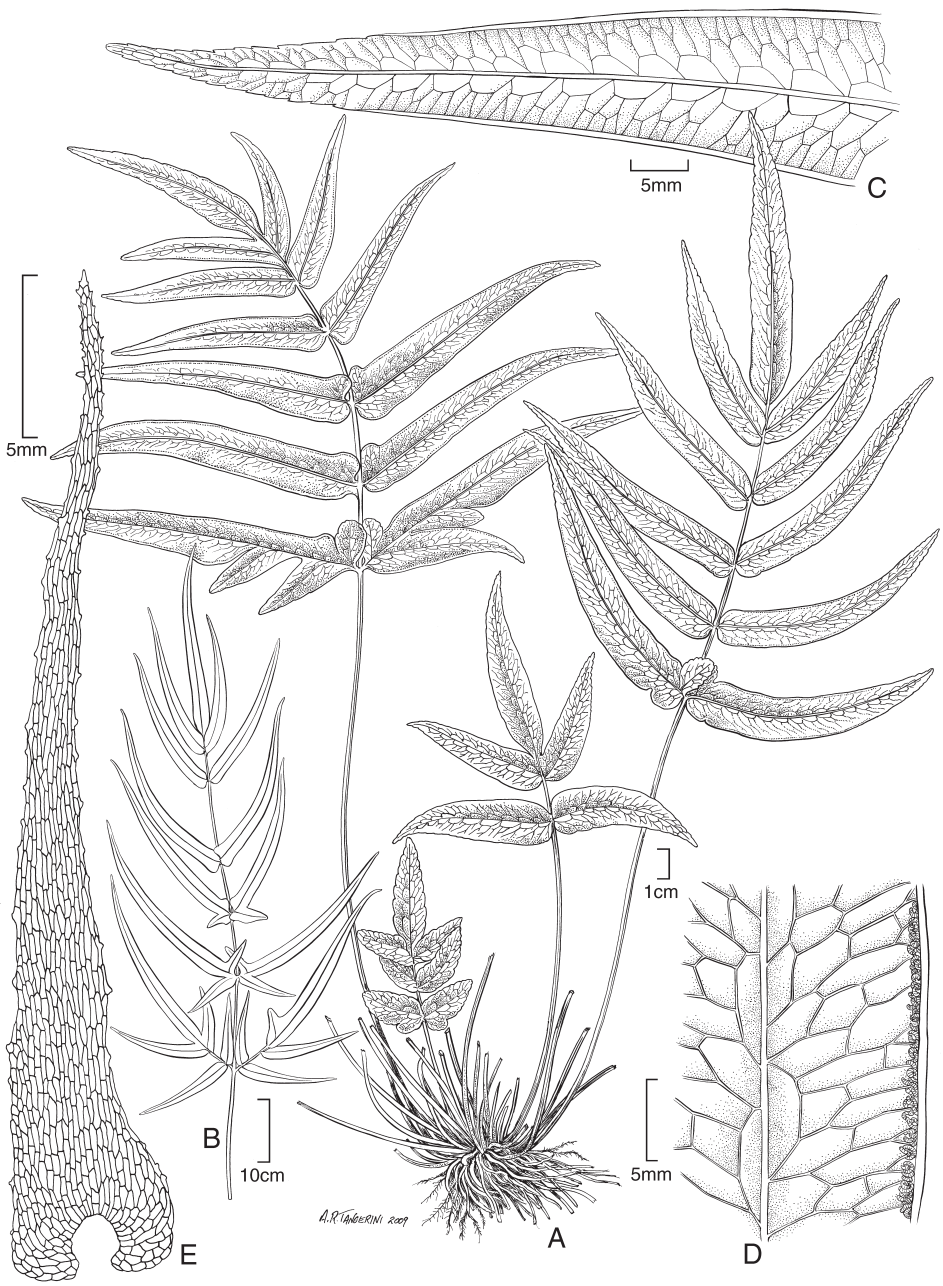
*Pteris tahuataensis* Lorence & K. R. Wood. **A** habit **B** blade **C** fertile pinna distal part **D** lower surface of fertile pinnule showing marginal sori **E** rhizome scale. Drawn from the type collection (Wood 10083) and field images.

###### Distribution.

Known only from Tahuata, Marquesas Islands.

###### Ecology.

This new terrestrial or lithophytic species occurs in wet forests and shrublands from about 418 to 914 m elevation, usually on windswept vertical cliffs in water seepage. A population of approximately 50–70 plants was observed at 914 m on windswept vertical cliffs around a natural spring on saturated basalt walls with *Leptochloa marquisensis* (F. Br.) P. M. Peterson & Judz. and *Selliguea feeioides* Copel., mosses, and lichens in wet forest of *Crossostylis biflora*, *Freycinetia* sp., *Metrosideros collina*, *Reynoldsia marchionensis*, *Weinmannia marquesana* var. *marquesana*, *Myrsine grantii*, and *Pandanus tectorius*. A second population occurs at 518 m in mesic forest with *Cerbera manghas* L., *Cyclophyllum barbatum*, *Hibiscus tiliaceus*, *Pandanus tectorius*, *Sapindus saponaria* L. and *Xylosma suaveolens* subsp. *pubigerum*. A third population is from lowland degraded mesic forest at 418 m elevation with *Cerbera manghas*, *Cyclophyllum barbatum*, *Ficus prolixa* var. *prolixa*, *Hibiscus tiliaceus*, *Pandanus tectorius*, and *Xylosma suaveolens* subsp. *pubigerum, Sapindus saponaria*. The main threats to this species include competition from invasive alien plant species and habitat destruction by feral goats and fire at lower elevations. Known only from three collections, the one population at 418 m having an estimated 50–70 plants (Wood 10085), the others were isolated plants.

###### Conservation status.

Proposed IUCN Red List Category **Critically Endangered** (CR): B2a, B2b i–iii; D): B2: total area of occupancy less than 10 km2 (ca. 5 km2); B2a, three populations known; b (i–iii), habitat continuing decline inferred; D, population estimated to number fewer than 250 mature individuals. The suitable habitat for *Pteris tahuataensis* on Tahuata (ca. 61 km2) is indicated as an endangered environment, threatened by human activity (deforestation, fire), feral animals, and invasive plants, reducing the extent of the forest. D, the rarity of this species is supported by the lack of collections and the small extant area, i.e. with only three known collections and an estimated total population size of fewer than 250 plants.

###### Etymology.

This new species is named for its only known island of occurrence.

###### Specimens examined.

**Marquesas Islands: Tahuata:** Hapatoni, village to the south of Vaitahu, ridge to summit above Patikoea point, UTM 0706136–8897872, 418 m, Wood 10085 (BISH, K, MO, NY, P, PAP, PTBG, UC, US); Amatea region, locations around Haaoiputeomo satellite dish, 9°92'S, 139°8'W, 914 m, Wood 10250 (PTBG, US).

### THELYPTERIDACEAE

With approximately 950 species (Smith et al. 2006) the Thelypteridaceae are one of the largest and most diverse fern families in the Marquesas and the tropics in general. Holttum recognized 15 genera in the Pacific and Australasia (Holttum 1976, 1977). However, delimitation of Holttum’s genera is often not clear-cut, and some of the most important characters he used for circumscribing them involve chromosome numbers and spore characters visible only at high magnification (e.g., under the scanning electron microscope). Furthermore, although many of Holttum’s genera seem monophyletic, a combination of characters is usually needed to circumscribe them. For these reasons, many authors now recognize only about five genera of Thelypteridaceae (Kramer and Green 1990; Smith and Cranfill 2002; Smith et al. 2006). For the purposes of the Marquesas Vascular Flora project we prefer to adopt this broader generic circumscription of *Thelypteris* Schmidel. and *Cyclosorus* Link, but follow Kramer and Green (1990) in recognizing the subgenera as natural groups. Two new species of *Cyclosorus* (subg. *Plesioneuron*) and one new species of *Thelypteris* (subg. *Coryphopteris*) have come to light among specimens collected in the Marquesas and are described below. One new combination is required, bringing the total number of Thelypteridaceae species in the Marquesas to nine. The genera may be separated using the characters in the following key.

Key to the genera of Thelypteridaceae in the Marquesas Islands

**Table:** 

1a	Veins to 14 pairs per segment; segments oblique, falcate; indusia absent or very small; sporangia generally setose	*Cyclosorus*
1b	Veins to 7 pairs per segment; segments spreading or slightly oblique, not falcate; indusia relatively large, persistent;sporangia lacking setae	*Thelypteris* (subg. *Coryphopteris*)

#### Cyclosorus

Viewed in the broad sense, *Cyclosorus* is the largest genus of Thelypteridaceae, comprising about 600 species in the tropics of both hemispheres. The seven indigenous or endemic and one naturalized species in the Marquesas may be separated by the following key.

Key to the species of *Cyclosorus* in the Marquesas Islands

**Table:** 

1a	Lower pinnae not reduced, lower 2–3 pairs usually not deflexed; veins all free; sinus with thickened membrane ± obliquely decurrent as a ridge almost to costa on lower surface	*Cyclosorus* (subg. *Plesioneuron*) *marquesicus*
1b	Lower pinnae not or only slightly reduced, lower 2 (−3) pairs deflexed; veins free or uniting, but sinus lacking thickened ridge decurrent toward costa on lower surface	2
2a	Blade with basal pinnae much narrowed on both sides near base, rudimentary pinnae below them 0−2 pairs, inconstant and irregular; blades with sessile spherical yellow glands present abaxially, especially along veins near segment tips; scales absent along costae abaxially, costal hairs ca. 0.1 mm long	*Cyclosorus* (subg. *Amphineuron*) *opulentus* (Kaulf.) Nakaike
2b	Blade with basal pinnae narrowed or not on both sides, but rudimentary basal pinnae lacking or several pairs regularly present; blades lacking sessile spherical yellow glands abaxially, or these spread ± evenly on laminar tissue; scales present or absent along costae abaxially, costal hairs 0.1−1 mm long, or hairs absent	3
3a	Fronds 1.5−3 m long; narrow, hairlike scales to 2 cm long present throughout stipe and on basal part of rachis, often breaking and leaving spine-like bases; lower surface of pinnae lacking sessile spherical yellow glands; sori exindusiate	*Cyclosorus* (subg. *Chingia*) *longissimus* (Brack.) Ching
3b	Fronds to 1.2 m long, usually smaller; stipe scales less than 2 cm long, these usually confined to base of stipe, not hairlike or leaving spine-like bases; sessile spherical yellow glands scattered all over lower surface of pinnae (except *Cyclosorus invisus*); sori indusiate	4
4a	Rhizomes creeping; laminar tissue between veins lacking sessile glands abaxially	5
4b	Rhizomes suberect or erect; laminar tissue between veins bearing numerous sessile, round, yellowish to orange-red glands abaxially	7
5a	Blades glabrous or very sparsely hairy abaxially, hairs if present less than 0.1 mm long on rachises and costae abaxially; costae glabrous adaxially; indusia absent; sporangia lacking hairs (Hiva Oa)	*Cyclosorus* (subg. *Pneumatopteris*) *florencei*
5b	Blades abundantly hairy abaxially, on costae, costules, veins, and between veins, hairs to 1 mm long; costae hairy adaxially; indusia present, hairy	6
6a	Rhizomes long-creeping; pinnae lobed 1/3−2/5 toward costae; veins and costules glabrous adaxially; sporangia bearing hairs (Fatu Hiva)	*Cyclosorus* (subg. *Sphaerstephanos*) *invisus* (G. Forst.) Copel.
6b	Rhizomes short-creeping; pinnae lobed 1/2−2/3 toward costae; veins and costules, sometimes also laminar tissue between veins, with acicular hairs adaxially; sporangia lacking hairs (Ua Huka)	*Cyclosorus* (subg. *Christella*) *dentatus* (Forssk.) Ching
7a	Abaxial costal hairs red-brown (castaneous), at least in part, curved toward pinna tips, some hairs > 0.5 mm long (Nuku Hiva)	*Cyclosorus* (subg. *Sphaerostephanos*) *castaneus*
7b	Abaxial costal hairs hyaline, spreading or ascending, straight or falcate, uniformly very short and < 0.1 mm long, or costae sometimes with scattered to rather dense longer hairs to ca. 1 mm; Society and Marquesas Islands (Fatu Hiva, Hiva Oa, Tahuata, Ua Huka)	*Cyclosorus* (subg. *Sphaerostephanos*) *subpectinatus* (Copel.) Ching

**Discussion.** *Cyclosorus subpectinatus* (Copel.) Ching, from the Marquesas and Society Islands, is extremely variable and possibly represents more than a single species, but the material at hand seems to intergrade. Specimens from different islands are all slightly different in various indument, venation, indusial, and size characteristics. Specimens with the largest indusia (to nearly 1 mm in diameter) and longest costal hairs are from Ua Huka (e.g., Dunn 340, BISH; Lorence 9307, PTBG, UC). The type of *Cyclosorus subpectinatus*, from Tahiti, also has rather long, dense costal hairs, but has smaller indusia only ca. 0.3 mm in diameter The sole collection seen from Tahuata (Wood 10270, P, PAP, PTBG, US) has exceptionally large fronds, with pinnae to 28 × 3.5 cm. Most specimens seen from both the Society Islands (Huahine, Moorea, Raiatea, and Tahiti), and the Marquesas Islands have relatively short, uniform costal hairs ca. 0.1 mm long and small indusia ca. 0.3 mm in diameter. Two specimens from Fatu Hiva (Chapin 789, BISH, cited by Holttum, 1977, as the sole specimen of the species from the Marquesas; and Florence 9509, BISH, P, US) seem to be nearly or quite exindusiate. All specimens seen of *Cyclosorus subpectinatus* have rather numerous to moderate, tan, adpressed, lanceolate costal scales abaxially, as well as dense, spherical, yellowish to orange-red sessile glands between the veins abaxially, but lack laminar glands adaxially.

##### 9. Cyclosorus (Sphaerostephanos) castaneus

A. R. Sm. & Lorence sp. nov.

urn:lsid:ipni.org:names:77112682-1

http://species-id.net/wiki/Cyclosorus_(Sphaerostephanos)_castaneus

Fig. 11 

###### Latin.

A Cyclosoro heterocarpo (Blume) Ching numerosis, nitidis, castaneis, falcatis pilis 0.3−1.5 mm longis super rachides, costis et costulis abaxialibus, parva indusia 0.4−0.6 mm diametro, magis marcatis aerophoribus, fortasseque longioribus, magisque bene-formatis caudicibus differt.

###### Type.

**Marquesas Islands:** Nuku Hiva: Toovii region, trail along ridge from near l’Economie Rurale complex to Ooumu peak, 860–1080 m, 17 July 1988, D. Lorence (with W.L. Wagner, J. Florence and S. Perlman) 6115 (holotype PTBG!; isotypes BISH!, PAP!, US!).

###### Description.

*Terrestrial ferns*; *rhizomes* erect, caudices 15–60 cm long; *fronds* clustered, 7–10 per rhizome; base of stipes to first large pinnae 40–50 cm, distal part of stipes bearing 7–10 pairs of reduced pinnae 3–5 cm apart, 1–7(–35) mm long, the largest ones sometimes trifid; *rachises* reddish brown, bearing numerous reddish, often curved hairs; proximal large pinnae narrowed at their bases; *blades*, excluding reduced proximal pinnae, 50–70 cm long; largest pinnae 11–16 × 1.5–2 cm, at their bases each with a swollen, tuberculiform aerophore (most developed and scalelike, to 4 mm long in young fronds), pinnae bases not auricled, apices caudate-acuminate, lobed 2/3–3/4 toward the costae (2–3 mm from costae), lobes slightly falcate, rounded at tips; costules ca. 3–5 mm apart; veins to ca. 8–9 pairs per segment, the basal pair from adjacent segments anastomosing and producing an excurrent vein 1–1.5 mm long to the sinus, the next acroscopic vein to sinus-membrane; abaxial surface of costae with very short, hyaline, spreading to distally curved hairs 0.1 mm long and much longer and stouter falcate hairs to 0.3–1.5 mm on costae, scales lacking or costae with a few adpressed, tan, amorphous scales to 2 × 0.1 mm; yellowish sessile glands borne on abaxial laminar surfaces, often dense, absent or very sparse on adaxial laminar surfaces; stout reddish, falcate hairs adaxially on costae and costules to 1 mm long, with short, falcate hairs ca. 0.2–0.3 mm long sometimes on veins and between veins, especially below sinuses. *Sori* medial; indusia reddish brown to tan, ca. 0.4–0.6 mm in diameter, glabrous or with a few short hairs 0.1–0.2 mm long; sporangia with sessile, yellow glands on the capsules.

**Figure 11.**
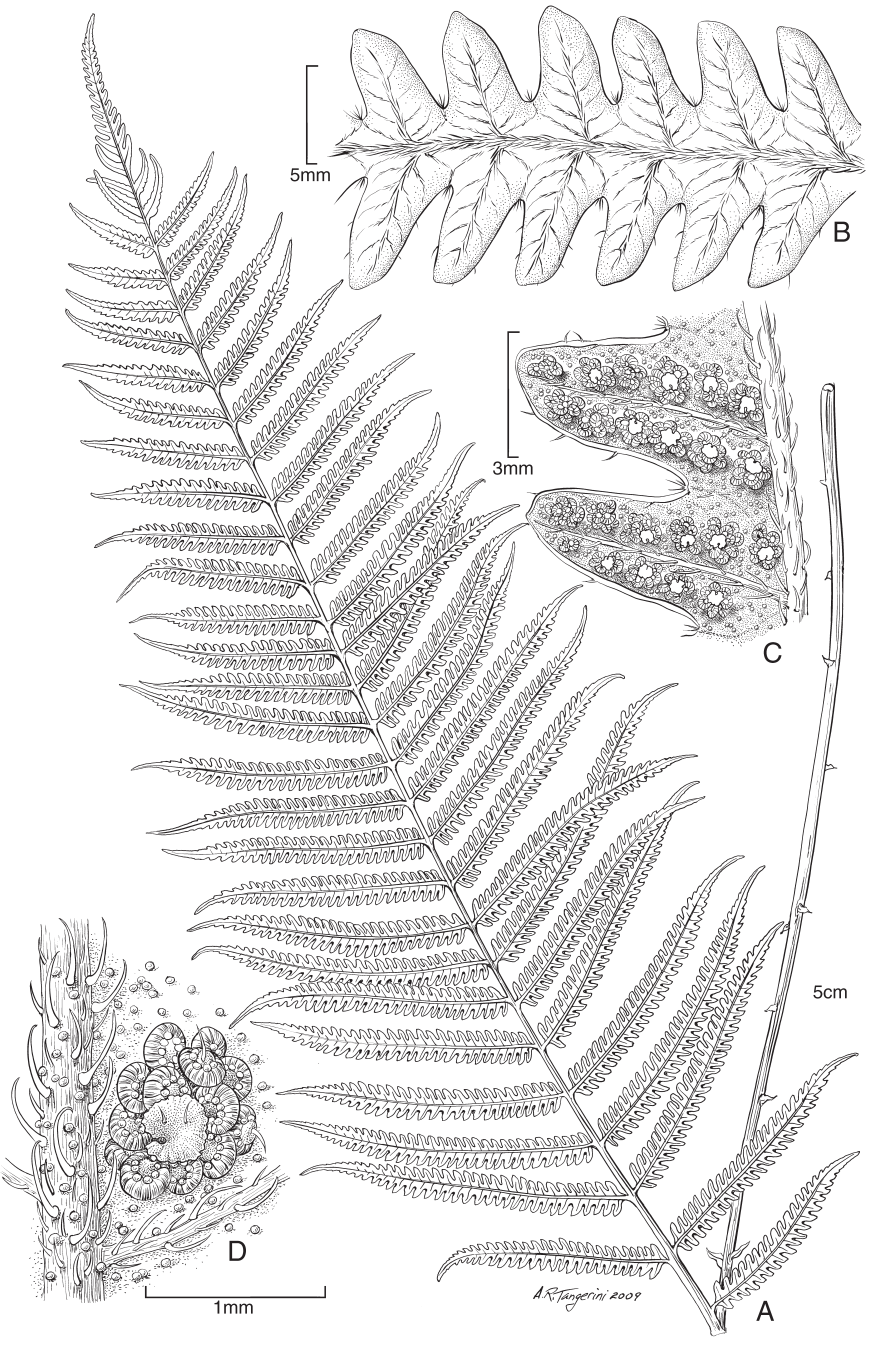
*Cyclosorus castaneus* A. R. Sm. & Lorence. **A** frond **B** upper surface of pinna **C** lower surface of fertile pinnules showing sori, hairs, and glands **D** detail of lower surface of fertile pinnule with sorus, hairs, and glands. Drawn from the type collection (Lorence et al. 6115).

###### Distribution.

Known only from Nuku Hiva, Marquesas Islands.

###### Ecology.

This new species occurs in montane rain forest with fern understory. All three specimens seen were collected on Nuku Hiva on or near Ooumu Peak in the Toovii area and probably represent a single population.

###### Conservation status.

Proposed IUCN Red List Category **Endangered** (EN): B2a, B2b i–iii): B2: total area of occupancy less than 500 km2 (ca. 50 km2). B2a, a single population known; b (i–iii), habitat continuing decline inferred. The suitable habitat for *Cyclosorus castaneus* on Nuku Huka (ca. 340 km2) is indicated as an endangered environment, threatened by human activity (deforestation), feral animals, and invasive plants, reducing the extent of the forest.

###### Etymology.

Named for the castaneous, curved hairs that are borne on the costae abaxially.

###### Specimens examined.

**Marquesas Islands: Nuku Hiva**: Toovii Plateau, trail behind L’Economie Rurale toward Ooumu peak; 3100 ft (945 m), S. Perlman 10125 (BISH, PTBG); Toovii, Ooumu area, top of Tapueahu Valley off new Hwy, 0851 S 14019 W, 3500–3700 ft (1067—1128 m), K. Wood 4578 (BISH, PAP, PTBG, US).

###### Discussion.

The three known specimens were previously determined as *Sphaerostephanos (Cyclosorus) heterocarpus* (Blume) Holttum, a related species known from Southeast Asia, Malesia, Australia (Queensland), Melanesia (Fiji, Vanuatu), and Samoa. From that species *Cyclosorus castaneus* differs in the numerous shiny, castaneous, falcate hairs 0.3–1.5 mm long, on the rachises, costae, and costules abaxially. These hairs vary in length, but are frequently longer than 0.5 mm on the costae. *Cyclosorus heterocarpus* shows considerable variation in indument (both length and disposition of hairs); however, the hairs in *Cyclosorus heterocarpus* are always hyaline, not so decidedly curved, and are generally much shorter than those in *Cyclosorus castaneus*. Holttum (1982) recognized many unnamed forms of *Cyclosorus heterocarpus* and produced a key to them based primarily on the depth of pinna lobing, presence/absence of sessile glands on the laminae adaxially, width of fertile pinnae, and presence/absence of hairs on the laminae adaxially. *Cyclosorus heterocarpus* also has larger indusia than *Cyclosorus castaneus*, often 1 mm in diameter or more, and the indusia envelop the sporangia when young. Some variants of *Cyclosorus heterocarpus* have sessile glands adaxially (rare in *Cyclosorus castaneous*) and less deeply incised pinnae (Holttum 1982). Aerophores in *Cyclosorus heterocarpus* appear to be much less developed, only a lunate, slightly raised area, or mammiform hump. In *Cyclosorus castaneus*, the aerophores are tuberculiform, peglike, or even scalelike, to 4 mm long. A single specimen from Ua Pou (Anakooma river valley just ESE of Oave peak, 470 m, Lorence 9117, PTBG) resembles *Cyclosorus castaneus* in having reddish hairs on the costae abaxially, but the hairs are less deeply colored and spreading, not falcate. Most sporangia seem empty, and what spores are formed are irregular, so it is possible this is a hybrid involving *Cyclosorus castaneus* and *Cyclosorus florencei*. However, neither parental species has been found on Ua Pou. Further study is clearly needed.

##### 10. Cyclosorus (Pneumatopteris) florencei

A. R. Sm. & Lorence sp. nov.

urn:lsid:ipni.org:names:77112683-1

http://species-id.net/wiki/Cyclosorus_(Pneumatopteris)_florencei

Fig. 12 

###### Latin.

A C. glandulifero (Brack.) Copel. absentibus falcatis, acicularibus pilis adaxialliter supra axiales costas et presentibus reductarum proximalium pinnarum paribus non tan numerosis (ca. 10 pro ca. 20), absentibus pilis supra sporangiales stipites et et receptacula differt.

###### Type.

**Marquesas Islands:** Hiva Oa: Atuona, trail to Hanamenu, 9°48'S, 139°04'W, wet forest, 29 Jul 1988,J. Florence (with D. Lorence, S. Perlman) 9598 (holotype BISH!; isotypes P, PAP, neither seen).

###### Description.

*Terrestrial ferns*; *rhizomes* creeping, to 10 mm in diameter; *fronds* spaced, base of stipes sparsely scaly, scales brown, lanceolate; stipes to ca. 70 cm long below first large pinnae, distal part of stipes to 9 mm in diameter at base, bearing ca. 10 pairs of greatly reduced pinnae 2.5–4 cm apart, these 1–10 mm long; proximal large pinnae narrowed at their bases; blades subcoriaceous, excluding reduced, glanduliform proximal pinnae, to ca. 60 cm long, gradually reduced distally to a pinnatifid apex; rachises tan to stramineous, glabrous; large developed pinnae to ca. 30 lateral pairs, to ca. 12 × 1.7 cm, at their bases each with a swollen, conical aerophore to ca. 0.5 mm long, pinnae bases not auricled, apices caudate-acuminate, lobed 2/5–1/2 toward the costae (3–4 mm from costae), lobes oblique and slightly falcate, subacute to rounded at tip; costules ca. 4 mm apart; veins to ca. 8–9  pairs per segment, prominent (especially abaxially) on both sides of laminae, the basal pair from adjacent segments generally obtusely united and producing an excurrent vein 2.5–3 mm long to the sinus, the next 1–1 1/2 pairs merging with this excurrent vein or running to a cartilaginous, raised, sinus membrane; abaxial surface of rachis, costae, costules, and veins nearly lacking hairs or with scattered, minute hairs less than 0.1 mm, costae bearing adpressed to slightly spreading, tan, amorphous (cell walls not readily discernible at 30×) scales to 2 × 0.2 mm; yellowish sessile glands absent on both laminar surfaces, pustules also lacking; hairs absent adaxially on costae, costules, and veins. *Sori* medial to supramedial; indusia absent; sporangia bearing yellowish capsular glands ca. 0.1 mm, lacking acicular hairs on sporangia and from receptacles.

**Figure 12.**
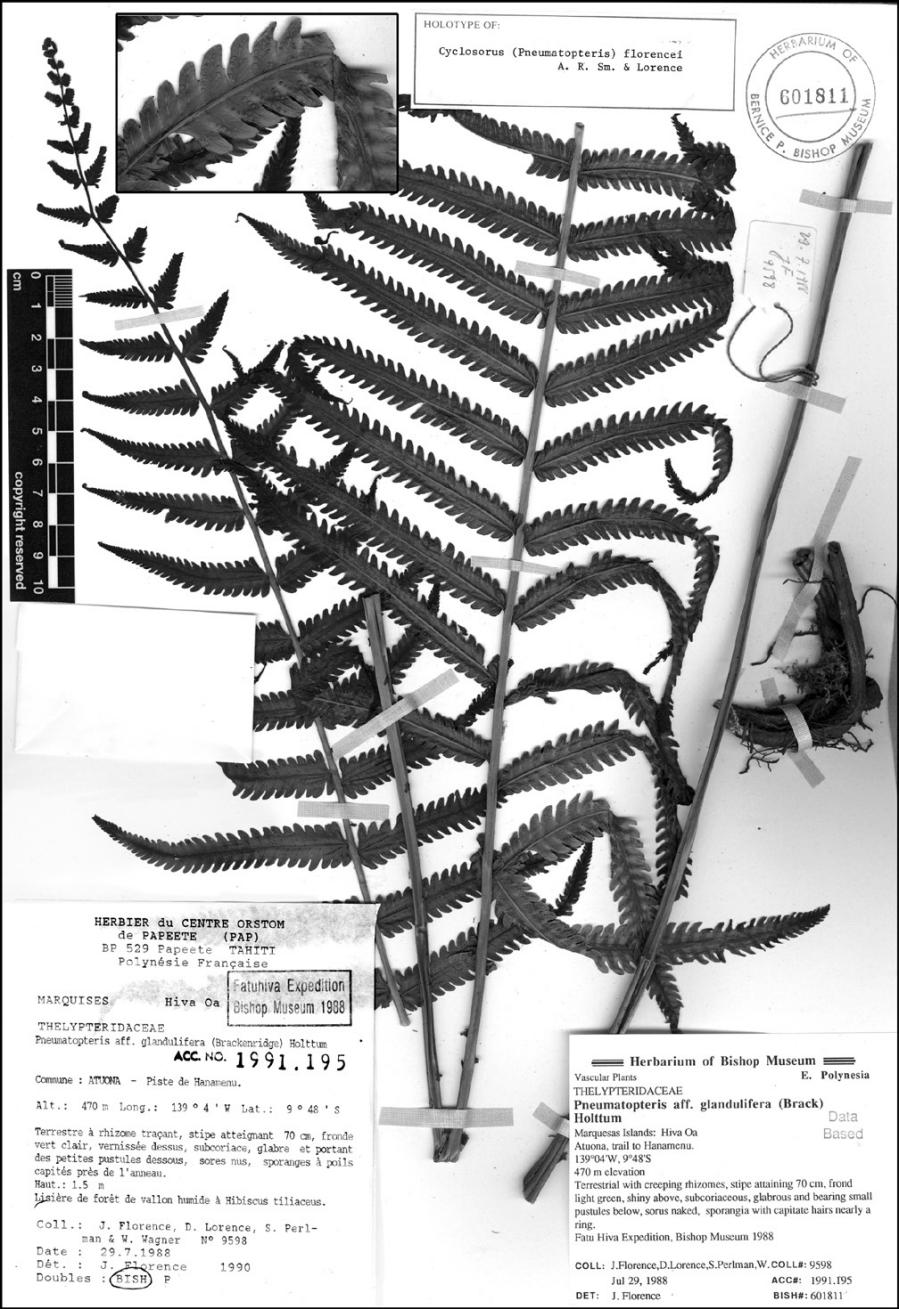
*Cyclosorus florencei* A. R. Sm. & Lorence Holotype collection with frond and part of rhizome, Florence et al. 9598 (BISH). Inset shows detail lower surface of fertile pinna with sori.

###### Distribution.

Hiva Oa, Marquesas Islands, known only from the type collection made along the trail from Atuona to Hanamenu.

###### Ecology.

Occurs in montane wet forest with *Crossostylis biflora*, *Freycinetia* sp., *Metrosideros collina*, *Weinmannia marquesana* var. *marquesana* and other species characteristic of this habitat.

###### Etymology.

We take pleasure in naming this new species for Jacques Florence (1951–) who has done so much to advance our knowledge of the flora of the Marquesas Islands and that of French Polynesia in general.

###### Conservation status.

Proposed IUCN Red List Category **Critically Endangered** (CR); B2a, B2b i–iii; D): B1, extent of occurrence estimated to be less than 100 km2; B2, area of occupancy estimated to be less than 10 km2 (ca. 9 km2), and B2a, a single population known; b (i–iii), habitat continuing decline inferred; D, population estimated to number fewer than 250 mature individuals. The suitable habitat for *Cyclosorus florencei* on Hiva Oa (ca. 315 km2) is indicated as an endangered environment, threatened by human activity (deforestation and fire), feral animals, and invasive plants, reducing the extent of the forest; and D, the rarity of this species is supported by the lack of collections and the small extant area within a commonly collected island, i.e. with a single collection.

###### Discussion.

This new species is perhaps most closely related to *Cyclosorus glanduliferus* (Brack.) Copel., from Rarotonga (Cook Islands), Samoa, Solomon Islands, and New Hebrides, and to *Cyclosorus stokesii* (E. D. Br.) Ching, known only from Rapa Iti in the Austral Islands (Holttum 1977).  The three species are similar in rhizome habit, blade dissection, and blade size. However, *Cyclosorus glanduliferus* differs from *Cyclosorus stokesii* in having more numerous glanduliform pinnae along the stipes (to about 20 pairs), longer aerophores at bases of costae, presence of small aerophores at costule bases, fewer and shorter costal scales, presence of numerous acicular, falcate hairs on the costae adaxially, and hairs borne on the sporangial stalks and receptacles. From *Cyclosorus stokesii*, *Cyclosorus florencei* differs in having less scaly stipe bases (scales numerous and ovate in *Cyclosorus stokesii*), fewer pairs of glanduliform proximal pinnae (to 20 pairs of glanduliform pinnae in *Cyclosorus stokesii*), lacking aerophores at bases of costules, more numerous, longer costal scales, 4–5 pairs of veins uniting below sinus or connivent at the sinuses, and lacking hairs from the sporangial stalks. Spore differences mentioned by Holttum (1977) between *Cyclosorus glanduliferus* and *Cyclosorus stokesii* cannot be evaluated for *Cyclosorus florencei* because the sporangia in the type of *Cyclosorus florencei* are immature. The label with the type indicates the presence of small pustules abaxially, but we fail to see any pustules of the kind often found in species of subg. *Pneumatopteris*; we do see numerous, slightly more reflective stomates (guard cells) at 30 times magnification. Until now no species of subg. *Pneumatopteris* has been recorded from the Marquesas Islands.

The following **new combination** in Thelypteridaceae is also required.

##### 11. Cyclosorus marquesicum

(Holttum) Lorence & A. R. Sm. comb. nov.

urn:lsid:ipni.org:names:77112686-1

http://species-id.net/wiki/Cyclosorus_marquesicum

Plesioneuron marquesicum Holttum, Allertonia 1: 192. 1977. [Basionym]

###### Type.

**Marquesas Islands:** Hivoa: Hana lafa, 700 m, October 1922, *W. B. Jones 1618* (Holotype BISH!).

###### Distribution.

Marquesas Islands (known from Hiva Oa, Tahuata, Ua Huka, and Ua Pou) and the Society Islands (Moorea). A list of exsiccatae is available on the Flora of the Marquesas website (http://botany.si.edu/pacificislandbiodiversity/marquesasflora/index.htm).

#### Thelypteris (subgen. Coryphopteris)

(Holttum (1976, 1977) circumscribed *Coryphopteris* Holttum as a genus of about 50 species with its greatest diversity in New Guinea and the Malesian region. However, we prefer to consider *Coryphopteris* as a subgeneric segregate of a more broadly circumscribed *Thelypteris* following the concepts of Kramer and Green (1990), Smith and Cranfill (2002), and Smith et al. (2006). For the purposes of the Marquesas Vascular Flora project we prefer to adopt two genera, *Thelypteris* and *Cyclosorus* in their broader sense, but follow Kramer and Green (1990) in recognizing the subgenera as natural groups.

Species belonging to *Thelypteris* subgenus *Coryphopteris* are small to medium-sized terrestrial ferns (rarely epiphytic) resembling miniature tree ferns with an erect or sometimes decumbent rhizome topped by a cluster of 1-pinnate fronds with the basal pinnae pairs deflexed in most species. Additional distinguishing characters include relatively large, sessile glands especially dense on the lower surface of the lamina, often abundant scales on the lower surface, and septate acicular hairs on the upper surface of the rachis and costa of some species. Most species belonging to subgenus *Coryphopteris* are restricted to low wet forest or mossy cloud forest habitat on high mountain ridge slopes and crests, often above 1,000 m elevation, where they grow in leached, nutrient poor soils (Holttum 1976).

Two species of *Thelypteris* subgenus *Coryphopteris* are known from the Marquesas, *Thelypteris quaylei* (E. D. Br.) Ching [*Coryphopteris quaylei* (E. D. Br.) Holttum], and a diminutive new species described below, *Thelypteris marquesensis*. *Thelypteris quaylei* is a larger, more robust species known from Nuku Hiva, Ua Pou, Ua Huka, Hiva Oa, and Fatu Hiva, generally at lower elevations (580–884 m). Certain collections of *Thelypteris quaylei* from the summit region of Ua Huka (700–884 m) tend to be relatively small, precociously fertile plants. However, the appearance and habitats of the two species are quite different and they can be separated morphologically by the characters in the following key.

Key to *Thelypteris* subg. *Corphyopteris* in the Marquesas Islands

**Table:** 

1a	Plants with stipes 1–5(–8.5) cm long, with scattered scales when young, with hairs to 0.5–0.7 mm long; scales near base of stipe 1-2 mm long; blades 2.5–14.5 cm long; pinnae 5–14 pairs; pinnae lobed 1/3–4/5 toward to costa, sessile glands lacking on surface, sori 1(–2) per segment; pinnule veins 1–5 pairs	*Thelypteris marquesensis*
1b	Plants with stipes 15–25(–33) cm long, densely scaly when young, glabrescent, with hairs to 0.2 mm long; scales near base of stipe 5–7 mm long; blades 20–30 cm long; pinnae (12–) 18–25 pairs; pinnae lobed 4/5–7/8 toward to costa, sessile glands present on surface, sori (1–) 3–4 per segment; pinnule veins 5–9 pairs	*Thelypteris quaylei*

##### 12. Thelypteris marquesensis

Lorence & K. R. Wood sp. nov.

urn:lsid:ipni.org:names:77112689-1

http://species-id.net/wiki/Thelypteris_marquesensis

Figs 13 15F 

###### Latin.

Species Thelypteri quayleii affinis, sed minori habitu, laminis cum 1-3 paribus inferis deminutisque pinnarum, et pinnarum abaxiali superficie sine sessilibus glandis differt.

###### Type.

**Marquesas Islands:** Hiva Oa; Temetiu, windswept ridges and drainages, 3280 ft [999 m], 9°48'S, 139°4'W, 26 August 1995, K. R. Wood 4408 (holotype PTBG-038499!; isotypes, P!, PAP!, US!).

###### Description.

*Small ferns*, usually lithophytic, forming colonies over wet basalt rock faces (rarely mossy tree trunks); *rhizomes* slender, decumbent to suberect, unbranched, radial, 1.5–7 cm long, 0.4–1.2 cm in diameter, densely clothed by stipe bases, sparsely covered in red-brown, unicellular acicular hairs; *fronds* 7–9 per rhizome; stipes 1–5(–8.5) cm long, medium brown, moderately covered in unicellular acicular, red-brown hairs 0.5–0.7 mm long and scales, the scales sparsely scattered, those near base ovate-falcate, red-brown to brownish black, 1–2 × 0.5–0.7 mm, margins entire or with sparse short projections; blades 1-pinnate-pinnatifid, 2.5–8.5 × 1.2–2.8 cm, oblong-elliptic, rachises medium to red-brown, moderately covered with light brown, curved acicular hairs 0.3–0.7 mm long, apex pinnatifid, pinnae 5–12 pairs, largest pinnae 0.6–1.4 × 0.3–0.5 cm, apex obtuse, lobed (1/3–)½ toward costa, lobes crenate, margins with acicular hairs 0.3–0.6 mm long, costules to 2 mm apart, lower pinnae slightly reduced and lower 2(–3) pairs deflexed, basal acroscopic pinnule 1 mm longer than next, veins 2–3 pairs in basal lobe, 1–2 pairs in middle lobes, abaxial surface of rachises and costae hirtellous with brown acicular hairs 0.2–0.7 mm long, adaxial surfaces glabrous or with occasional short acicular hairs 0.2–0.5 mm, sessile glands absent. *Sori* medial, 1(–2) per segment; indusia reniform to subcircular, margins with sessile glands, otherwise glabrous.

**Figure 13.**
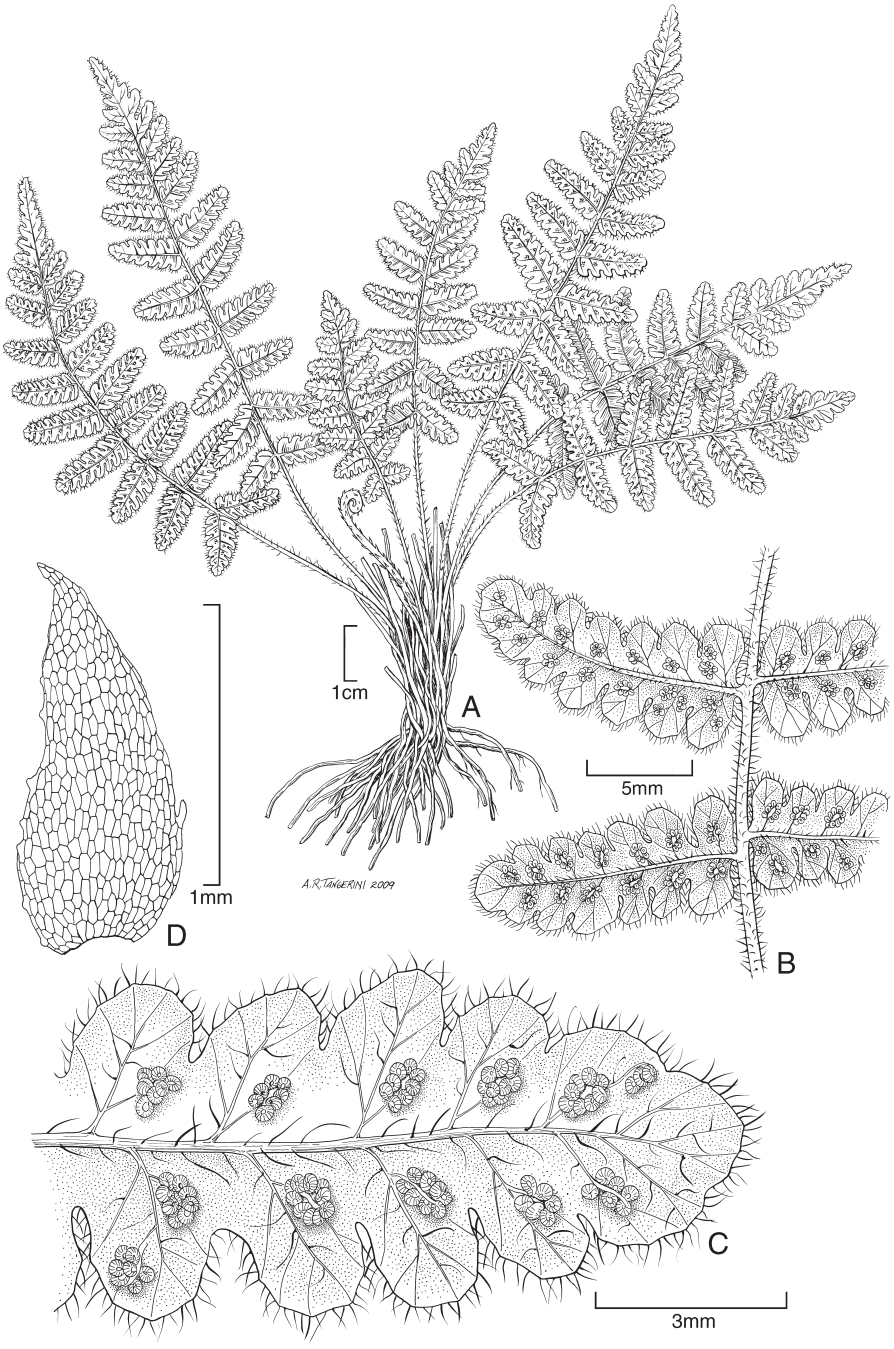
*Thelypteris marquesensis* Lorence & K. R. Wood. **A** habit **B** lower surface fertile pinnae showing sori **C** lower surface of fertile pinna showing sori and hairs **D** rhizome scale. Drawn from the type collection (Wood 4408) and field images.

**Figure 14.**
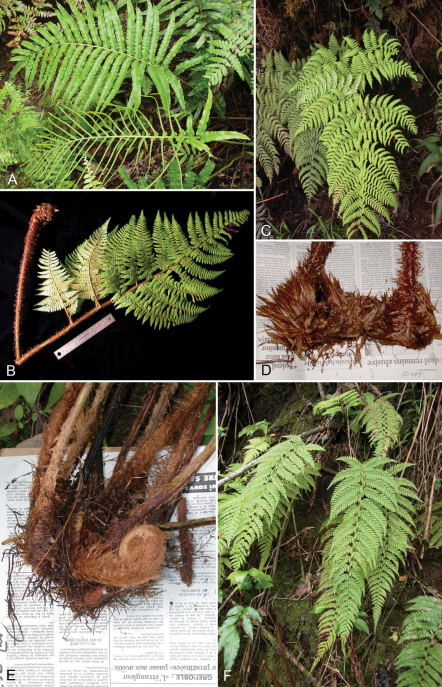
**A** *Blechnum pacificum*, habit, habit, fertile and sterile fronds (Fatu Hiva, Lorence 6171) **B–D** *Dryopteris macropholis* **B** frond (Ua Huka, Wood 10489, type) **C** habit, D rhizome and stipe bases (**C, D** Hiva Oa, Wood 10045) **E** *Polystichum kenwoodii*, rhizome and stipe bases (Hiva Oa, Wood 10232) **F** *Polystichum uahukaense*, habit (Ua Huka, Wood 10552).

**Figure 15.**
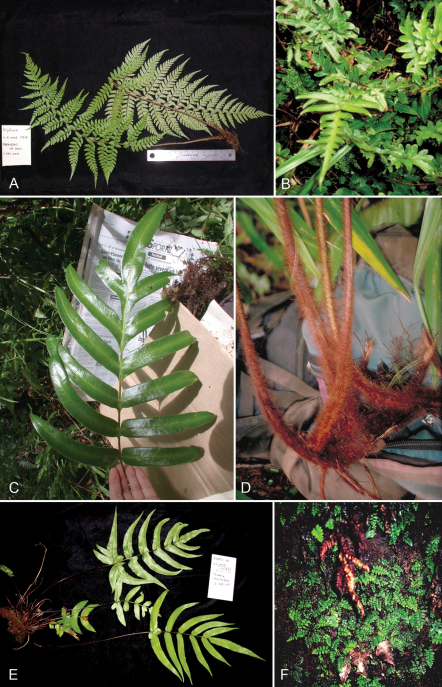
**A** *Polystichum uahukaense*, frond (Ua Huka, Wood 10518) **B** *Pteris hivaoaensis* (Hiva Oa, Wood 4374) **C, D** *Pteris marquesensis* **C** frond **D** rhizome and stipe bases (both Hiva Oa, Wood 10871) **E** *Pteris tahuataensis* (Tahuata, Wood 10083) **F** *Thelypteris marquesensis*, habit, colony on wet rock face (Hiva Oa, Wood 4408).

###### Distribution.

Known only on Hiva Oa, Marquesas Islands, from a single population in the vicinity of Mt. Temetiu, on the island’s central summit crest.

###### Ecology.

*Thelypteris marquesensis* occurs at 1000–1200 m elevation in low, windswept, montane wet forests and shrublands with *Alsophila tahitensis*, *Cheirodendron bastardianum*, *Crossostylis biflora*, *Cyrtandra* spp., *Freycinetia* spp.*, Leptochloa marquisensis*, *Melicope* spp., *Metrosideros collina*, *Psychotria* spp.*, Weinmannia marquesana* var. *marquesana*, and abundant pteridophytes in the understory. This new species is rare and localized, although in one area it occurs in dense colonies of hundreds of plants that completely cover wet banks and rock faces in and around shallow depressions or grottos, with fronds often appressed to the rock face (K. Wood, pers. obs.). Threats to this species include rooting by feral pigs and invasion by aggressive alien plant species, notably *Elephantopus mollis*, *Psidium guajava*, and *Syzygium cumini*.

###### Etymology.

This new species is named for the Marquesas Islands, where it is known currently known only from Hiva Oa.

###### Conservation status.

Proposed IUCN Red List Category **Critically Endangered** (CR): B2a, B2b i–iii): B1, extent of occurrence estimated to be less than 100 km2; B2, area of occupancy estimated to be less than 10 km2 (ca. 9 km2), and B2a, a single population known; b (i–iii), habitat continuing decline inferred. The suitable habitat for *Thelypteris marquesensis* on Hiva Oa (ca. 315 km2), confined to Mt. Temetiu and vicinity, is indicated as an endangered environment, threatened by human activity (deforestation and fire), feral animals (pigs), and invasive plants, reducing the extent of the forest.

###### Discussion.

This new species differs from *Thelypteris quaylei* in its much smaller habit (although some specimens of *Thelypteris quaylei* from the Ua Huka summit are atypically small), fronds with 1–3, gradually reduced basal pairs of pinnae, and lack of sessile glands on the lamina surfaces (but present on indusial margins), a feature it shares with *Thelypteris fasciculata* Ching from New Caledonia, New Guinea, and the Celebes (Holttum 1977). Although *Thelypteris marquesensis* may occur in the vicinity of *Thelypteris quaylei* (Lorence et al. 8942, PTBG), the latter species tends to grow in wet forest understory either terrestrially or on mossy boulders, although it is sometimes found in shady depressions on steep ridge slopes either as individuals or forming small colonies.

###### Specimens examined.

**Marquesas Islands:** Hiva Oa: Temetiu, windswept ridges and drainages, 3900 ft, 9°48'S, 139°4'W, Wood 4392 (P, PAP, PTBG, UC, US); chemin d’Atuona a Hanamenu par Feani, pente vers Hanamenui, Schäfer 5195 (US); Atuona–Feani trail, crest of Feani ridge, Sachet & Decker1127 (US), *1192* (US).

## Supplementary Material

XML Treatment for Blechnum pacificum

XML Treatment for Dryopteris macropholis

XML Treatment for Dryopteris sweetorum

XML Treatment for Polystichum kenwoodii

XML Treatment for Polystichum uahukaense

XML Treatment for Pteris hivaoaensis

XML Treatment for Pteris marquesensis

XML Treatment for Pteris tahuataensis

XML Treatment for Cyclosorus (Sphaerostephanos) castaneus

XML Treatment for Cyclosorus (Pneumatopteris) florencei

XML Treatment for Cyclosorus marquesicum

XML Treatment for Thelypteris marquesensis
